# Supplementary material for: Metagenomic Detection of Divergent Insect- and Bat-Associated Viruses in Plasma from Two African Individuals Enrolled in Blood-Borne Surveillance
Source: Viruses. 2023 Apr 21;15(4):1022. doi: 10.3390/v15041022 (PMC10145552; doi:10.3390/v15041022)
Supplement: Supplementary file 1 [file viruses-15-01022-s001.zip › viruses-2332003-supplementary.pdf]

Supplementary information for:

Metagenomic detection of divergent insect- and bat-associated viruses in plasma from two African individuals enrolled in blood-borne surveillance

Gregory S. Orf[1,2], Ana Olivo[1,2], Barbara Harris[1,2], Sonja L. Weiss[1,2], Asmeeta Achari[2,3], Guixia Yu[2,3], Scot Federman[2,3], Dora Mbanya[4], Linda James[5], Samuel Mampunza[5], Charles Y. Chiu[2,3,6], Mary A. Rodgers[1,2], Gavin A. Cloherty[1,2], and Michael G. Berg[1,2,*]

1 Infectious Disease Research, Abbott Diagnostics, Abbott Park, IL, USA
2 Abbott Pandemic Defense Coalition
3 Department of Laboratory Medicine, University of California-San Francisco, San Francisco, CA, USA
4 Faculty of Medicine and Biomedical Sciences, University of Yaoundé I, Yaoundé, Cameroon
5 School of Medicine, Université Protestante au Congo, Kinshasa, DRC
6 Department of Medicine, University of California-San Francisco, San Francisco, CA, USA
* Correspondence: E-mail: michael.berg@abbott.com; Tel.: +1-224-668-0742

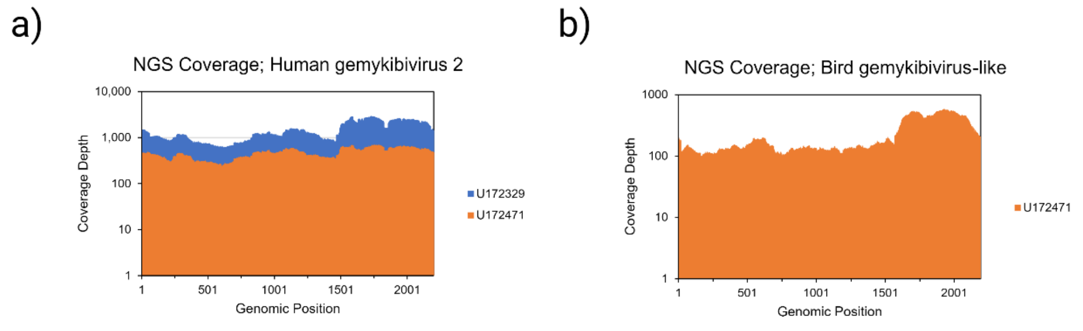

Figure S1. Genomic coverage statistics for the assembled gemycircularviruses (**a**) human gemykibivirus 2 and (**b**) bird-like gemykibivirus. The genomic coordinates are in the same orientation as the maps shown in the Main Text Figure 2.

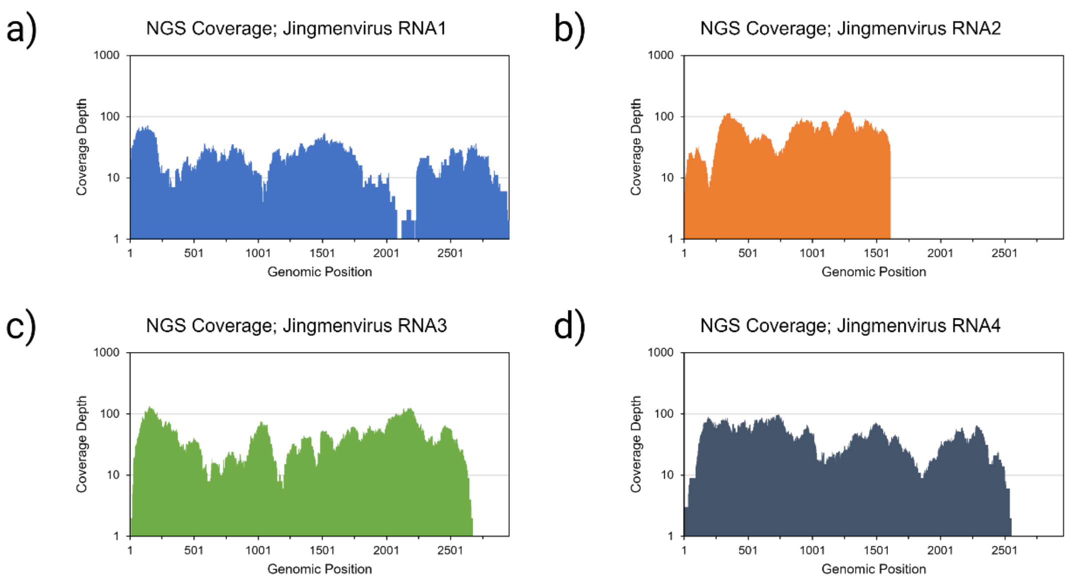

Figure S2. Genomic coverage statistics for the assembled Jingmanvirus; segments 1-4 (**a**-**d**). The genomic coordinates are in the same orientation as the maps shown in the Main Text Figure 2.

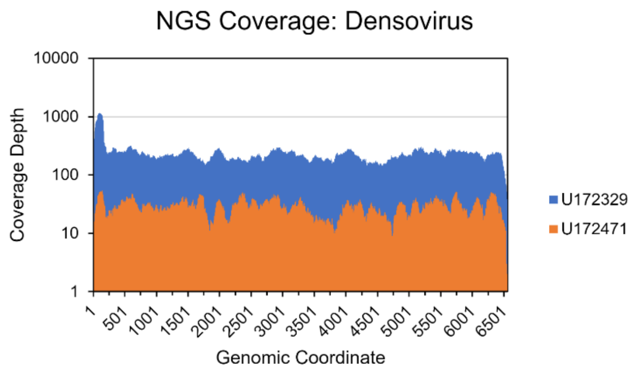

Figure S3. Genomic coverage statistics for the assembled densovirus. The genomic coordinates are in the same orientation as the maps shown in the Main Text Figure 2.

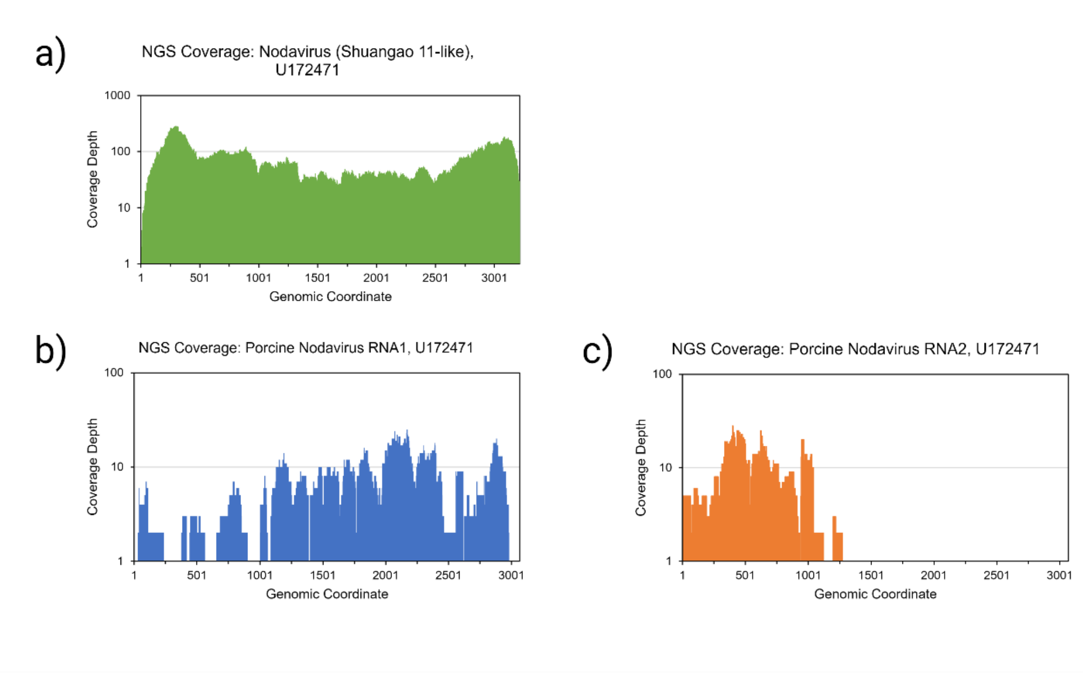

Figure S4. Genomic coverage statistics for the assembled nodaviruses: Shuangao 11-like virus (**a**) and Porcine nodavirus (**b-c**). The genomic coordinates are in the same orientation as the maps shown in the Main Text Figure 2.

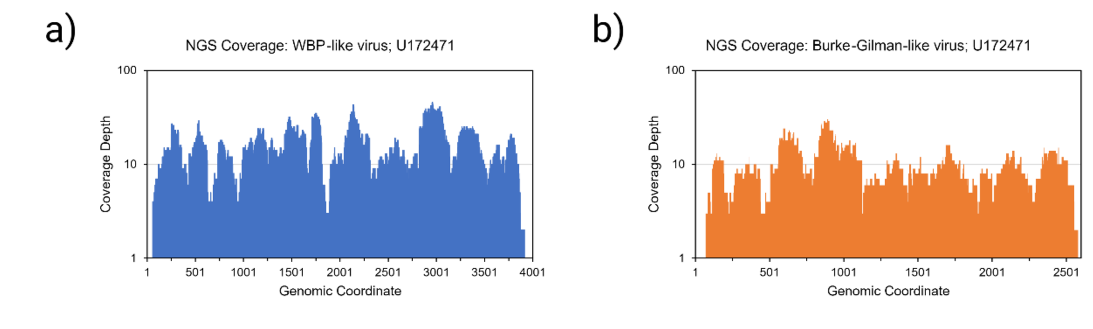

Figure S5. Genomic coverage statistics for the partially-assembled picornaviruses: Washington bat-like virus (**a**) and Burke-Gilman-like virus (**b**). The genomic coordinates relate to the assembled contig, not to the expected genome coordinate based on homology to other picornaviruses.

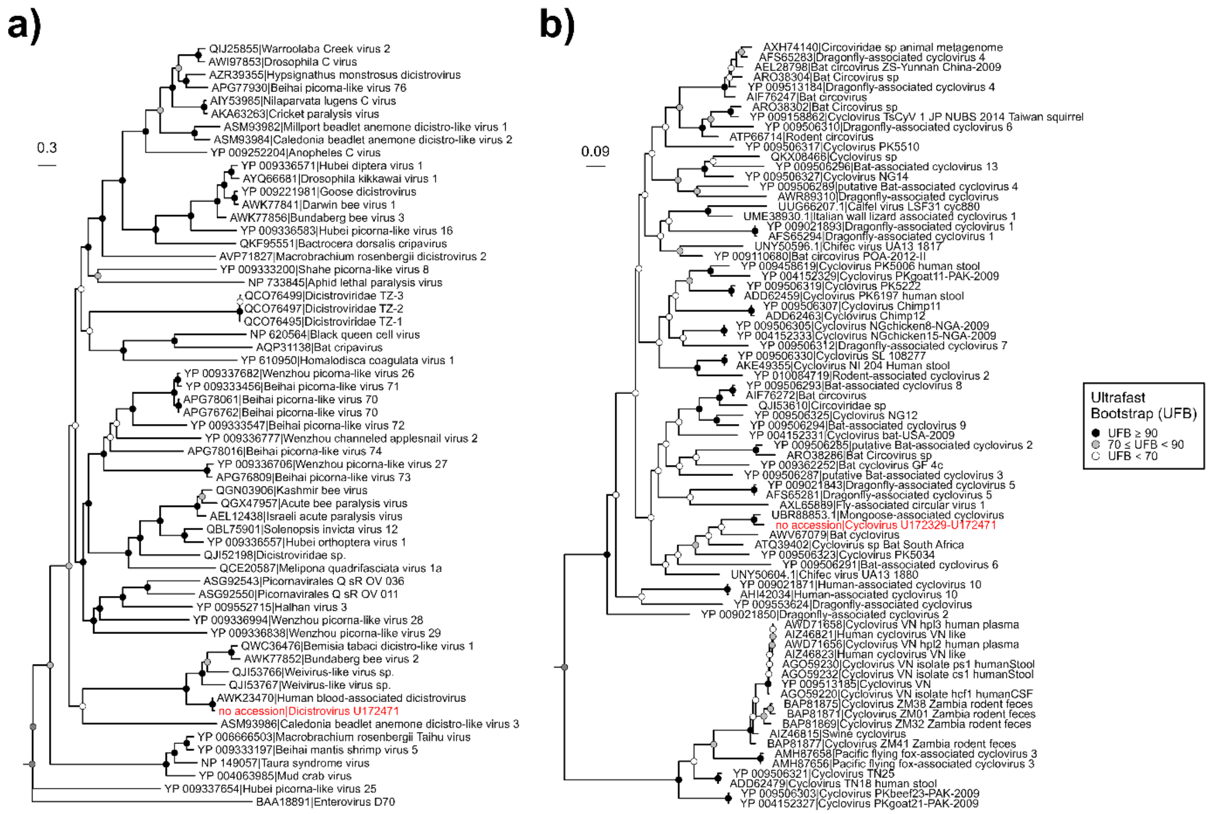

Figure S6. Maximum likelihood phylogenetic reconstruction of the ORF1 protein sequence of the dicistroviruses (**a**) and the Rep protein sequence of cycloviruses (**b**). Each tree is midpoint rooted and branch lengths are drawn to scale in terms of substitutions per site with the scale indicated in each panel. The input multiple sequence alignments were produced using the program MAFFT, with the E-INS-i algorithm used in panel (**a**) and the L-INS-i algorithm used in panel (**b**). Branching supports from 1000 replicates of Ultrafast Bootstrap (UFB) are shown as symbols at the nodes, with accompanying legend shown at right.

Table S1. List of accessions and metadata referring for the reference sequences from the phylum Pisuviricota utilized as a training set for the linear discriminant analysis shared in the Main Text, Figure 5a.

ACCESSION	ORGANISM	STRAIN	HOST	SEGMENT	CLASS	ORDER	FAMILY	HOST RANGE
**NC_002548**	Acute bee paralysis virus	NA	Apis mellifera	NA	Pisoniviricetes	Picornavirales	Dicistroviridae	Invertebrates
**NC_038762**	Aeonium ringspot virus	Aeonium	Aeonium sp.	RNA1	Pisoniviricetes	Picornavirales	Secoviridae	Plants
**NC_038761**	Aeonium ringspot virus	NA	Aeonium sp.	RNA2	Pisoniviricetes	Picornavirales	Secoviridae	Plants
**NC_026470**	African bat icavirus PREDICT-06105	PREDICT-06105	Hipposideros gigas	NA	Pisoniviricetes	Picornavirales	Picornaviridae	Vertebrates
**NC_043537**	African eggplant mosaic virus	2013-281	Solanum aethiopicum	NA	Stelpaviricetes	Patatavirales	Potyviridae	Plants
**NC_034207**	African eggplant yellowing virus	eMA4	Solanum aethiopicum	NA	Pisoniviricetes	Sobelivirales	Solemoviridae	Plants
**NC_005903**	Agropyron mosaic virus	ND402	NA	NA	Stelpaviricetes	Patatavirales	Potyviridae	Plants
**NC_001918**	Aichi virus 1	A846/88	Homo sapiens	NA	Pisoniviricetes	Picornavirales	Picornaviridae	Vertebrates
**NC_004421**	Aichivirus B	U-1	NA	NA	Pisoniviricetes	Picornavirales	Picornaviridae	Vertebrates
**NC_055161**	Aimelvirus 1	gpai001	NA	NA	Pisoniviricetes	Picornavirales	Picornaviridae	Unknown
**NC_035198**	Aimelvirus 2	gpai002	NA	NA	Pisoniviricetes	Picornavirales	Picornaviridae	Unknown
**NC_029993**	Alfalfa enamovirus 1	Manfredi	alfalfa	NA	Pisoniviricetes	Sobelivirales	Solemoviridae	Plants
**NC_010736**	Algerian watermelon mosaic virus	H4	NA	NA	Stelpaviricetes	Patatavirales	Potyviridae	Plants
**NC_046964**	Alphacoronavirus Bat-CoV/P.kuhlii/Italy /3398-19/2015	Bat-CoV/P.kuhlii/Italy /3398-19/2015	Pipistrellus kuhlii	NA	Pisoniviricetes	Nidovirales	Cornidovirineae	Vertebrates
**NC_055493**	Alpinia oxyphylla mosaic virus	Alpo	Alpinia oxyphylla	NA	Stelpaviricetes	Patatavirales	Potyviridae	Plants
**NC_006553**	anativirus A1	TW90A	NA	NA	Pisoniviricetes	Picornavirales	Picornaviridae	Vertebrates
**NC_038744**	Andean potato mottle virus	C	NA	NA	Pisoniviricetes	Picornavirales	Secoviridae	Plants
**NC_030115**	Anopheles C virus	Ngousso	Anopheles coluzzii	NA	Pisoniviricetes	Picornavirales	Dicistroviridae	Invertebrates
**NC_030232**	Antarctic picorna-like virus 1	APLV1	unknown	NA	Pisoniviricetes	Picornavirales	environmental	Unknown
**NC_030233**	Antarctic picorna-like virus 2	APLV2	unknown	NA	Pisoniviricetes	Picornavirales	environmental	Unknown
**NC_030234**	Antarctic picorna-like virus 3	APLV3	unknown	NA	Pisoniviricetes	Picornavirales	environmental	Unknown
**NC_030235**	Antarctic picorna-like virus 4	APLV4	unknown	NA	Pisoniviricetes	Picornavirales	environmental	Unknown
**NC_023483**	Antheraea pernyi iflavirus	LnApIV-02	Antheraea pernyi	NA	Pisoniviricetes	Picornavirales	Iflaviridae	Invertebrates
**NC_004365**	Aphid lethal paralysis virus	NA	NA	NA	Pisoniviricetes	Picornavirales	Dicistroviridae	Invertebrates
**NC_034973**	Aphis glycines virus 3	S6-IA	Aphis glycines	NA	Pisoniviricetes	Picornavirales	Dicistroviridae	Invertebrates
**NC_035115**	Apis dicistrovirus	AWD-1151	Apis mellifera	NA	Pisoniviricetes	Picornavirales	Dicistroviridae	Invertebrates
**NC_014905**	Apium virus Y	Ce	Apium graveolens	NA	Stelpaviricetes	Patatavirales	Potyviridae	Plants
**NC_003787**	Apple latent spherical virus	NA	NA	RNA 1	Pisoniviricetes	Picornavirales	Secoviridae	Plants
**NC_003788**	Apple latent spherical virus	NA	NA	RNA 2	Pisoniviricetes	Picornavirales	Secoviridae	Plants
**NC_043411**	Apricot latent ringspot virus	Modesto	NA	NA	Pisoniviricetes	Picornavirales	Secoviridae	Plants
**NC_006057**	Arabis mosaic virus	Neustadt an der Weinstrasse (NW)	grapevine 'Pinot gris'	RNA 1	Pisoniviricetes	Picornavirales	Secoviridae	Plants
**NC_006056**	Arabis mosaic virus	NW	grapevine cultivar Pinot gris	RNA 2	Pisoniviricetes	Picornavirales	Secoviridae	Plants
**NC_055501**	Areca palm necrotic ringspot virus	XC1	Areca catechu (palm)	NA	Stelpaviricetes	Patatavirales	Potyviridae	Plants
**NC_040836**	Areca palm necrotic spindle-spot virus	HNBT	areca palm	NA	Stelpaviricetes	Patatavirales	Potyviridae	Plants
**NC_036585**	Armigeres iflavirus	10P38-310	Armigeres spp.	NA	Pisoniviricetes	Picornavirales	Iflaviridae	Invertebrates
**NC_018176**	Arracacha mottle virus	C-17	Arracacia xanthorrhiza	NA	Stelpaviricetes	Patatavirales	Potyviridae	Plants
**NC_020898**	Arracacha virus B	PV-0082	Oxalis tuberosa	RNA 1	Pisoniviricetes	Picornavirales	Secoviridae	Plants
**NC_020897**	Arracacha virus B	oca	Oxalis tuberosa	RNA 2	Pisoniviricetes	Picornavirales	Secoviridae	Plants
**NC_017914**	Artemisia virus A	NA	Artemisia annua	NA	Pisoniviricetes	Sobelivirales	Solemoviridae	Plants
**NC_043685**	Artichoke Italian latent virus	AILV-V	NA	RNA 2	Pisoniviricetes	Picornavirales	Secoviridae	Plants
**NC_043684**	Artichoke Italian latent virus	AILV-V	NA	RNA 1	Pisoniviricetes	Picornavirales	Secoviridae	Plants
**NC_026759**	Artichoke latent virus	FR37	artichoke; cv. Camus de Bretagne	NA	Stelpaviricetes	Patatavirales	Potyviridae	Plants
**NC_038862**	Artichoke yellow ringspot virus	NA	NA	NA	Pisoniviricetes	Picornavirales	Secoviridae	Plants
**NC_025821**	Asparagus virus 1	DSMZ PV-0954	NA	NA	Stelpaviricetes	Patatavirales	Potyviridae	Plants
**NC_024489**	Asterionellopsis glacialis RNA virus	AglaRNAV	NA	NA	Pisoniviricetes	Picornavirales	Marnaviridae	Chromista

**NC_043416**	Astrovirus dogfaeces/Italy/2005	44625	NA	NA	Stelpaviricetes	Stellavirales	Astroviridae	Vertebrates
**NC_027426**	Astrovirus Er/SZAL6/HUN/2011	Er/SZAL6/HUN/2011	Coracias garrulus (European roller)	NA	Stelpaviricetes	Stellavirales	Astroviridae	Vertebrates
**NC_011400**	Astrovirus MLB1	MLB1	NA	NA	Stelpaviricetes	Stellavirales	Astroviridae	Vertebrates
**NC_016155**	Astrovirus MLB2	MLB2/human/Stl/WD0559/2008	Homo sapiens	NA	Stelpaviricetes	Stellavirales	Astroviridae	Vertebrates
**NC_019028**	Astrovirus MLB3	MLB3/human/Vellore/26564/2004	Homo sapiens	NA	Stelpaviricetes	Stellavirales	Astroviridae	Vertebrates
**NC_013060**	Astrovirus VA1	VA1	Homo sapiens	NA	Stelpaviricetes	Stellavirales	Astroviridae	Vertebrates
**NC_019026**	Astrovirus VA3	VA3/human/Vellore/28054/2005	Homo sapiens	NA	Stelpaviricetes	Stellavirales	Astroviridae	Vertebrates
**NC_019027**	Astrovirus VA4	VA4/human/Nepal/S5363/2008	Homo sapiens	NA	Stelpaviricetes	Stellavirales	Astroviridae	Vertebrates
**NC_016896**	Astrovirus wild boar/WBAstV-1/2011/HUN	wild boar/WBAstV-1/2011/HUN	Sus scrofa (wild boar)	NA	Stelpaviricetes	Stellavirales	Astroviridae	Vertebrates
**NC_024031**	Atlantic salmon calicivirus	Nordland/2011	Salmo salar L. (Atlantic salmon)	NA	Pisoniviricetes	Picornavirales	Caliciviridae	Vertebrates
**NC_003990**	tremovirus A1	Calnek	NA	NA	Pisoniviricetes	Picornavirales	Picornaviridae	Vertebrates
**NC_001451**	Infectious bronchitis virus	Beaudette	NA	NA	Pisoniviricetes	Nidovirales	Cornidovirineae	Vertebrates
**NC_028970**	Avisivirus Pf-CHK1/AsV	Pf-CHK1/AsV	Gallus gallus domesticus (domestic chicken)	NA	Pisoniviricetes	Picornavirales	Picornaviridae	Vertebrates
**NC_034206**	kunsagivirus C1	baboon/M27-KuV/1986/TAN	Papio cynocephalus	NA	Pisoniviricetes	Picornavirales	Picornaviridae	Vertebrates
**NC_024709**	Ball python nidovirus 1	07-53	Python regius	NA	Pisoniviricetes	Nidovirales	Tornidovirineae	Vertebrates
**NC_009745**	Banana bract mosaic virus	NA	banana	NA	Stelpaviricetes	Patatavirales	Potyviridae	Plants
**NC_030847**	Barbacena virus Y	KLL097	weed	NA	Stelpaviricetes	Patatavirales	Potyviridae	Plants
**NC_003483**	Barley mild mosaic virus	UK-F	barley	RNA 1	Stelpaviricetes	Patatavirales	Potyviridae	Plants
**NC_003482**	Barley mild mosaic virus	UK-F	barley	RNA 2	Stelpaviricetes	Patatavirales	Potyviridae	Plants
**NC_029906**	Barley virus G	Gimje	barley	NA	Pisoniviricetes	Sobelivirales	Solemoviridae	Plants
**NC_039035**	Barley yellow dwarf virus GPV	05YC3	NA	NA	Pisoniviricetes	Sobelivirales	Solemoviridae	Plants
**NC_043124**	Barley yellow dwarf virus SGV	SGV	NA	NA	Pisoniviricetes	Sobelivirales	Solemoviridae	Plants
**NC_002990**	Barley yellow mosaic virus	Yancheng	barley	RNA 1	Stelpaviricetes	Patatavirales	Potyviridae	Plants
**NC_002991**	Barley yellow mosaic virus	Yancheng	barley	RNA 2	Stelpaviricetes	Patatavirales	Potyviridae	Plants
**NC_009741**	Basella rugose mosaic virus	AC	Anredera cordifolia (Madeira vine)	NA	Stelpaviricetes	Patatavirales	Potyviridae	Plants
**NC_035758**	Bastrovirus 7	NA	Homo sapiens	NA	Stelpaviricetes	Stellavirales	Astroviridae	Unknown
**NC_032426**	Bastrovirus-like_virus/VietNam/Bat/17819_21	17819_21	Bat	NA	Stelpaviricetes	Stellavirales	Astroviridae	Unknown
**NC_035471**	Bastrovirus/VietNam/Bat/16715_78	16715_78	Bat	NA	Stelpaviricetes	Stellavirales	Astroviridae	Unknown
**NC_032423**	Bastrovirus/VietNam/Porcine/17489_85	17489_85	porcine	NA	Stelpaviricetes	Stellavirales	Astroviridae	Unknown
**NC_032484**	Bastrovirus/VietNam/Rat/16715_10	16715_10	rat	NA	Stelpaviricetes	Stellavirales	Astroviridae	Unknown
**NC_038368**	Bat astrovirus Hp/Guangxi/LC03/2007	LC03	Hipposideros pomona	NA	Stelpaviricetes	Stellavirales	Astroviridae	Vertebrates
**NC_043103**	Bat astrovirus Tm/Guangxi/LD38/2007	LD38	Taphozous melanopogon	NA	Stelpaviricetes	Stellavirales	Astroviridae	Vertebrates
**NC_043098**	Bat astrovirus Tm/Guangxi/LD71/2007	LD71	Taphozous melanopogon	NA	Stelpaviricetes	Stellavirales	Astroviridae	Vertebrates
**NC_043100**	Bat astrovirus Tm/Guangxi/LD77/2007	LD77	Taphozous melanopogon	NA	Stelpaviricetes	Stellavirales	Astroviridae	Vertebrates
**NC_010437**	Bat coronavirus 1A	AFCD62	NA	NA	Pisoniviricetes	Nidovirales	Cornidovirineae	Vertebrates
**NC_014470**	Bat coronavirus BM48-31/BGR/2008	BtCoV/BM48-31/BGR/2008	Rhinolophus blasii	NA	Pisoniviricetes	Nidovirales	Cornidovirineae	Vertebrates
**NC_022103**	Bat coronavirus CDPHE15/USA/2006	bat/USA/CDPHE15/2006	Myotis lucifugus	NA	Pisoniviricetes	Nidovirales	Cornidovirineae	Vertebrates
**NC_034440**	Bat coronavirus	PREDICT/PDF-2180	Pipistrellus cf. hesperidus	NA	Pisoniviricetes	Nidovirales	Cornidovirineae	Vertebrates
**NC_048212**	Bat coronavirus	CMR704-P12	Eidolon helvum	NA	Pisoniviricetes	Nidovirales	Cornidovirineae	Vertebrates
**NC_033824**	Bat dicibavirus	NA	Eidolon helvum	NA	Pisoniviricetes	Picornavirales	Marnaviridae	Unknown
**NC_038316**	Bat hepatovirus BUO2BF86Colafr2010	BUO2BF86Colafr2010	Coleura afra	NA	Pisoniviricetes	Picornavirales	Picornaviridae	Vertebrates
**NC_038313**	Bat hepatovirus SMG18520Minmav2014	SMG18520Minmav2014	Miniopterus cf. manavi	NA	Pisoniviricetes	Picornavirales	Picornaviridae	Vertebrates
**NC_025217**	Bat Hp-betacoronavirus/Zhejiang2013	Zhejiang2013	Hipposideros pratti	NA	Pisoniviricetes	Nidovirales	Cornidovirineae	Vertebrates

**NC_033823**	Bat iflavirus	NA	Eidolon helvum	NA	Pisoniviricetes	Picornavirales	Iflaviridae	Unknown
**NC_015940**	Bat picornavirus 1	NC16A	Miniopterus pusillus	NA	Pisoniviricetes	Picornavirales	Picornaviridae	Unknown
**NC_015941**	Bat picornavirus 2	MH9F	Miniopterus magnater	NA	Pisoniviricetes	Picornavirales	Picornaviridae	Unknown
**NC_015934**	Bat picornavirus 3	TLC5F	Rhinolophus sinicus	NA	Pisoniviricetes	Picornavirales	Picornaviridae	Unknown
**NC_043071**	Bat picornavirus	BtMr-PicoV/JX2010	Myotis ricketti	NA	Pisoniviricetes	Picornavirales	Picornaviridae	Vertebrates
**NC_043072**	Bat picornavirus BatPV/V1/13 Hun	BatPV/V1/13/Hun	Miniopterus schreibersii	NA	Pisoniviricetes	Picornavirales	Picornaviridae	Vertebrates
**NC_033820**	Bat sapelovirus	NA	Eidolon helvum	NA	Pisoniviricetes	Picornavirales	Picornaviridae	Vertebrates
**NC_033776**	Bat sapovirus	Bat-SaV/Limbe65/CAM/2014	Eidolon helvum (Straw-coloured fruit bat)	NA	Pisoniviricetes	Picornavirales	Caliciviridae	Vertebrates
**NC_017936**	Bat sapovirus TLC58/HK	TLC58/HK	bat	NA	Pisoniviricetes	Picornavirales	Caliciviridae	Vertebrates
**NC_004047**	Bean common mosaic necrosis virus	Michigan	Phaseolus vulgaris	NA	Stelpaviricetes	Patatavirales	Potyviridae	Plants
**NC_003397**	Bean common mosaic virus	R	cowpea	NA	Stelpaviricetes	Patatavirales	Potyviridae	Plants
**NC_003496**	Bean pod mottle virus	KY G-7	NA	RNA 1	Pisoniviricetes	Picornavirales	Secoviridae	Plants
**NC_003495**	Bean pod mottle virus	KY G-7	NA	RNA 2	Pisoniviricetes	Picornavirales	Secoviridae	Plants
**NC_028139**	Bean rugose mosaic virus	Parana	Glycine max (soybean)	RNA 1	Pisoniviricetes	Picornavirales	Secoviridae	Plants
**NC_028146**	Bean rugose mosaic virus	Parana	Glycine max (soybean)	RNA 2	Pisoniviricetes	Picornavirales	Secoviridae	Plants
**NC_003492**	Bean yellow mosaic virus	MB4	NA	NA	Stelpaviricetes	Patatavirales	Potyviridae	Plants
**NC_002766**	Beet chlorosis virus	BChV-2a	NA	NA	Pisoniviricetes	Sobelivirales	Solemoviridae	Plants
**NC_003491**	Beet mild yellowing virus	2ITB	NA	NA	Pisoniviricetes	Sobelivirales	Solemoviridae	Plants
**NC_005304**	Beet mosaic virus	BtMV-Wa	Beta vulgaris L.; red beet	NA	Stelpaviricetes	Patatavirales	Potyviridae	Plants
**NC_003693**	Beet ringspot virus	S	NA	RNA 1	Pisoniviricetes	Picornavirales	Secoviridae	Plants
**NC_003694**	Beet ringspot virus	S	NA	RNA 2	Pisoniviricetes	Picornavirales	Secoviridae	Plants
**NC_004756**	Beet western yellows virus	USA	NA	NA	Pisoniviricetes	Sobelivirales	Solemoviridae	Plants
**NC_055601**	Begonia flower breaking virus	YN-Tiger	Begonia bowerae cv. Tiger	NA	Stelpaviricetes	Patatavirales	Potyviridae	Plants
**NC_032490**	Beihai hermit crab virus 4	BHJJX25971	hermit crab	NA	Pisoniviricetes	Nidovirales	Ronidovirineae	Invertebrates
**NC_032496**	Beihai Nido-like virus 1	BZL87871	Turritella sea snails	NA	Pisoniviricetes	Nidovirales	Mesnidovirineae	Invertebrates
**NC_032492**	Beihai Nido-like virus 2	BHXun32263	Charybdis crab	NA	Pisoniviricetes	Nidovirales	Ronidovirineae	Invertebrates
**NC_027915**	Bellflower vein chlorosis virus	CT1	Campanula takesimana	NA	Pisoniviricetes	Picornavirales	Secoviridae	Plants
**NC_039002**	Bellflower veinal mottle virus	SW	Campanula takesimana	NA	Stelpaviricetes	Patatavirales	Potyviridae	Plants
**NC_046956**	Bellinger River virus	J248	Myuchelys georgesi (Bellinger River snapping turtle)	NA	Pisoniviricetes	Nidovirales	Tornidovirineae	Vertebrates
**NC_010646**	Beluga whale coronavirus SW1	SW1	Delphinapterus leucas (beluga whale)	NA	Pisoniviricetes	Nidovirales	Cornidovirineae	Vertebrates
**NC_043212**	Berne virus	isolate P138/72	NA	RNA 1	Pisoniviricetes	Nidovirales	Tornidovirineae	Vertebrates
**NC_043211**	Berne virus	isolate P138/72	NA	RNA 2	Pisoniviricetes	Nidovirales	Tornidovirineae	Vertebrates
**NC_038294**	Betacoronavirus England 1	H123990006	Homo sapiens	NA	Pisoniviricetes	Nidovirales	Cornidovirineae	Vertebrates
**NC_039207**	Betacoronavirus Erinaceus/VMC/DEU/2012	ErinaceusCoV/2012-174/GER/2012	Erinaceus europaeus	NA	Pisoniviricetes	Nidovirales	Cornidovirineae	Vertebrates
**NC_026011**	Betacoronavirus HKU24	HKU24-R05005I	Rattus norvegicus (Norway rat)	NA	Pisoniviricetes	Nidovirales	Cornidovirineae	Vertebrates
**NC_023014**	Bidens mosaic virus	SP01	Bidens pilosa	NA	Stelpaviricetes	Patatavirales	Potyviridae	Plants
**NC_014325**	Bidens mottle virus	SF-1	Helianthus annuus	NA	Stelpaviricetes	Patatavirales	Potyviridae	Plants
**NC_035184**	Big Sioux River virus	Kenya P9	Aphis fabae	NA	Pisoniviricetes	Picornavirales	Dicistroviridae	Invertebrates
**NC_032840**	Biomphalaria virus 3	NA	Biomphalaria glabrata	NA	Pisoniviricetes	Picornavirales		Invertebrates
**NC_032112**	Bivalve RNA virus G1	G1	NA	NA	Pisoniviricetes	Picornavirales	Dicistroviridae	Invertebrates
**NC_032114**	Bivalve RNA virus G3	G3	NA	NA	Pisoniviricetes	Picornavirales		Invertebrates
**NC_032115**	Bivalve RNA virus G5	G5	NA	NA	Pisoniviricetes	Picornavirales	Dicistroviridae	Invertebrates
**NC_003784**	Black queen cell virus	South African	Apis mellifera	NA	Pisoniviricetes	Picornavirales	Dicistroviridae	Invertebrates
**NC_008182**	Black raspberry necrosis virus	BRDaV-1	NA	RNA 1	Pisoniviricetes	Picornavirales	Secoviridae	Plants
**NC_008183**	Black raspberry necrosis virus	BRDaV-1	NA	RNA 2	Pisoniviricetes	Picornavirales	Secoviridae	Plants
**NC_008558**	Blackberry virus Y	3	blackberry	NA	Stelpaviricetes	Patatavirales	Potyviridae	Plants
**NC_003502**	Blackcurrant reversion virus	NA	Ribes nigrum L.	RNA 2	Pisoniviricetes	Picornavirales	Secoviridae	Plants

**NC_003509**	Blackcurrant reversion virus	NA	NA	RNA 1	Pisoniviricetes	Picornavirales	Secoviridae	Plants
**NC_019415**	Blue squill virus A	SW3	NA	NA	Stelpaviricetes	Patatavirales	Potyviridae	Plants
**NC_038764**	Blueberry latent spherical virus	NA	NA	1	Pisoniviricetes	Picornavirales	Secoviridae	Plants
**NC_038763**	Blueberry latent spherical virus	NA	NA	2	Pisoniviricetes	Picornavirales	Secoviridae	Plants
**NC_029578**	Blueberry shoestring virus	BSSV	Vaccinium sp.	NA	Pisoniviricetes	Sobelivirales	Solemoviridae	Plants
**NC_018506**	limnipivirus A1	04-032	Lepomis macrochirus	NA	Pisoniviricetes	Picornavirales	Picornaviridae	Vertebrates
**NC_027713**	Bombyx mori iflavirus	BMI1	Bombyx mori strain Kinsyu-Syowa	NA	Pisoniviricetes	Picornavirales	Iflaviridae	Invertebrates
**NC_038305**	cardiovirus C1	BCV-1	Rattus norvegicus	NA	Pisoniviricetes	Picornavirales	Picornaviridae	Vertebrates
**NC_055538**	Botrylloides leachii nidovirus	SRR2729873	Botrylloides leachii	NA	Pisoniviricetes	Nidovirales	Mesnidovirineae	Invertebrates
**NC_043096**	Bottlenose dolphin astrovirus 1	Bd1	Tursiops truncatus (Bottlenose dolphin)	NA	Stelpaviricetes	Stellavirales	Astroviridae	Vertebrates
**NC_023632**	Bovine astrovirus B170/HK	B170/HK	cattle	NA	Stelpaviricetes	Stellavirales	Astroviridae	Vertebrates
**NC_023631**	Bovine astrovirus B18/HK	B18/HK	cattle	NA	Stelpaviricetes	Stellavirales	Astroviridae	Vertebrates
**NC_023630**	Bovine astrovirus B76-2/HK	B76-2/HK	cattle	NA	Stelpaviricetes	Stellavirales	Astroviridae	Vertebrates
**NC_023629**	Bovine astrovirus B76/HK	B76/HK	cattle	NA	Stelpaviricetes	Stellavirales	Astroviridae	Vertebrates
**NC_024498**	Bovine astrovirus CH13	CH13	cattle	NA	Stelpaviricetes	Stellavirales	Astroviridae	Vertebrates
**NC_024297**	Bovine astrovirus	BAstV-GX7/CHN/2014	cattle	NA	Stelpaviricetes	Stellavirales	Astroviridae	Vertebrates
**NC_030793**	Bovine calicivirus strain Kirklareli	Kirklareli	bovine	NA	Pisoniviricetes	Picornavirales	Caliciviridae	Vertebrates
**NC_003045**	Bovine coronavirus	BCoV-ENT	NA	NA	Pisoniviricetes	Nidovirales	Cornidovirineae	Vertebrates
**NC_001859**	Enterovirus E	VG-5-27	NA	NA	Pisoniviricetes	Picornavirales	Picornaviridae	Vertebrates
**NC_018668**	hunnivirus A1	BHUV1/2008/HUN	Bos taurus (cattle)	NA	Pisoniviricetes	Picornavirales	Picornaviridae	Vertebrates
**NC_027199**	Bovine nidovirus TCH5	TCH5	Bos taurus	NA	Pisoniviricetes	Nidovirales	Tornidovirineae	Vertebrates
**NC_026249**	Bovine picornavirus	TCH6	Bos taurus	NA	Pisoniviricetes	Picornavirales	Picornaviridae	Vertebrates
**NC_038303**	Bovine rhinitis A virus	Sd-1	bovine	NA	Pisoniviricetes	Picornavirales	Picornaviridae	Vertebrates
**NC_010354**	bovine rhinitis B virus 1	EC11	bovine	NA	Pisoniviricetes	Picornavirales	Picornaviridae	Vertebrates
**NC_034245**	Bovine rhinovirus 1	SD-1	calf	NA	Pisoniviricetes	Picornavirales	Picornaviridae	Vertebrates
**NC_040586**	Brassica napus RNA virus 1	SP2S	Brassica napus	NA	Pisoniviricetes	Picornavirales	Secoviridae	Plants
**NC_016038**	Brassica yellows virus	BrYV-ABJ	Brassica napus var. napobrassica	NA	Pisoniviricetes	Sobelivirales	Solemoviridae	Plants
**NC_007447**	Breda virus	Breda 1	NA	NA	Pisoniviricetes	Nidovirales	Tornidovirineae	Vertebrates
**NC_009530**	Brevicoryne brassicae virus - UK	NA	Brevicoryne brassicae	NA	Pisoniviricetes	Picornavirales	Iflaviridae	Invertebrates
**NC_022004**	Broad bean true mosaic virus	EV-11	NA	RNA 1	Pisoniviricetes	Picornavirales	Secoviridae	Plants
**NC_022006**	Broad bean true mosaic virus	EV-11	NA	RNA 2	Pisoniviricetes	Picornavirales	Secoviridae	Plants
**NC_005289**	Broad bean wilt virus 1	ATCC PV132	NA	RNA 1	Pisoniviricetes	Picornavirales	Secoviridae	Plants
**NC_005290**	Broad bean wilt virus 1	ATCC PV132	NA	RNA 2	Pisoniviricetes	Picornavirales	Secoviridae	Plants
**NC_003003**	Broad bean wilt virus 2	ME	NA	RNA 1	Pisoniviricetes	Picornavirales	Secoviridae	Plants
**NC_003004**	Broad bean wilt virus 2	ME	NA	RNA 2	Pisoniviricetes	Picornavirales	Secoviridae	Plants
**NC_038560**	Broad-leafed dock virus A	ab032 Auckland	Rumex obtusifolius	NA	Stelpaviricetes	Patatavirales	Potyviridae	Plants
**NC_003501**	Brome streak mosaic virus	NA	NA	NA	Stelpaviricetes	Patatavirales	Potyviridae	Plants
**NC_020105**	Brugmansia mosaic virus	SK	Brugmansia suaveolens	NA	Stelpaviricetes	Patatavirales	Potyviridae	Plants
**NC_014536**	Brugmansia suaveolens mottle virus	Bs-Campinas	NA	NA	Stelpaviricetes	Patatavirales	Potyviridae	Plants
**NC_028811**	BtMr-AlphaCoV/SAX2011	BtMr-SAX2011	Myotis ricketti	NA	Pisoniviricetes	Nidovirales	Cornidovirineae	Vertebrates
**NC_028833**	BtNv-AlphaCoV/SC2013	BtNv-SC2013	Nyctalus velutinus	NA	Pisoniviricetes	Nidovirales	Cornidovirineae	Vertebrates
**NC_028814**	BtRf-AlphaCoV/HuB2013	BtRf-HuB2013	Rhinolophus ferrumequinum	NA	Pisoniviricetes	Nidovirales	Cornidovirineae	Vertebrates
**NC_028824**	BtRf-AlphaCoV/YN2012	BtRf-YN2012	Rhinolophus ferrumequinum	NA	Pisoniviricetes	Nidovirales	Cornidovirineae	Vertebrates
**NC_011547**	Bulbul coronavirus HKU11-934	NA	red-whiskered bulbul	NA	Pisoniviricetes	Nidovirales	Cornidovirineae	Vertebrates

**NC_018572**	Caladenia virus A	KP1	Caladenia latifolia	NA	Stelpaviricetes	Patatavirales	Potyviridae	Plants
**NC_006875**	Calicivirus isolate TCG	TCG 14	bovine	NA	Pisoniviricetes	Picornavirales	Caliciviridae	Vertebrates
**NC_012699**	Calicivirus pig/AB90/CAN	AB90	Sus scrofa (swine)	NA	Pisoniviricetes	Picornavirales	Caliciviridae	Vertebrates
**NC_034975**	California sea lion astrovirus 2	CSL2	Zalophus californianus (California sea lion)	NA	Stelpaviricetes	Stellavirales	Astroviridae	Vertebrates
**NC_030794**	Callistephus mottle virus	DJ	Callistephus chinensis	NA	Stelpaviricetes	Patatavirales	Potyviridae	Plants
**NC_028752**	Camel alphacoronavirus	camel/Riyadh/Ry141/2015	camel	NA	Pisoniviricetes	Nidovirales	Cornidovirineae	Vertebrates
**NC_046965**	Canada goose coronavirus	Cambridge_Bay_2017	Branta canadensis	NA	Pisoniviricetes	Nidovirales	Cornidovirineae	Vertebrates
**NC_026814**	Canine astrovirus	Gillingham/2012/UK	NA	NA	Stelpaviricetes	Stellavirales	Astroviridae	Vertebrates
**NC_004542**	Canine vesivirus	NA	NA	NA	Pisoniviricetes	Picornavirales	Caliciviridae	Vertebrates
**NC_034971**	Canine kobuvirus	SMCD-59	dog	NA	Pisoniviricetes	Picornavirales	Picornaviridae	Vertebrates
**NC_016964**	Canine picornavirus	325	Canis familiaris	NA	Pisoniviricetes	Picornavirales	Picornaviridae	Vertebrates
**NC_013261**	Canna yellow streak virus	NA	Canna	NA	Stelpaviricetes	Patatavirales	Potyviridae	Plants
**NC_023422**	Caprine kobuvirus	12Q108	black goat	NA	Pisoniviricetes	Picornavirales	Picornaviridae	Vertebrates
**NC_039088**	Cardamom mosaic virus	KS	Elettaria cardamomum	NA	Stelpaviricetes	Patatavirales	Potyviridae	Plants
**NC_023162**	Carp picornavirus 1	F37/06	Cyprinus carpio	NA	Pisoniviricetes	Picornavirales	Picornaviridae	Vertebrates
**NC_038320**	Carrot necrotic dieback virus	Anthriscus	Daucus carota	NA	Pisoniviricetes	Picornavirales	Secoviridae	Plants
**NC_006265**	Carrot red leaf virus	UK-1	NA	NA	Pisoniviricetes	Sobelivirales	Solemoviridae	Plants
**NC_025254**	Carrot thin leaf virus	CTLV-Cs	Coriandrum sativum	NA	Stelpaviricetes	Patatavirales	Potyviridae	Plants
**NC_025479**	Carrot torradovirus 1	CTV-1_RNA1_H6	Daucus carota	RNA 1	Pisoniviricetes	Picornavirales	Secoviridae	Plants
**NC_025480**	Carrot torradovirus 1	CTV-1_RNA2_H6	Daucus carota	RNA 2	Pisoniviricetes	Picornavirales	Secoviridae	Plants
**NC_012698**	Cassava brown streak virus	KOR6	cassava	NA	Stelpaviricetes	Patatavirales	Potyviridae	Plants
**NC_023986**	Casuarina virus	71	Coquillettidia xanthogaster	NA	Pisoniviricetes	Nidovirales	Mesnidovirineae	Invertebrates
**NC_027210**	Catharanthus mosaic virus	Mandevilla-US	Mandevilla sp. 'Sunmandeho' Sun Parasol Giant White	NA	Stelpaviricetes	Patatavirales	Potyviridae	Plants
**NC_015668**	Cavally virus	C79	Aedes harrisoni	NA	Pisoniviricetes	Nidovirales	Mesnidovirineae	Invertebrates
**NC_055507**	Celery latent virus	Ag097	Apium graveolens Secalinum Group	NA	Stelpaviricetes	Patatavirales	Potyviridae	Plants
**NC_015393**	Celery mosaic virus	California	Apium graveolens L. (celery)	NA	Stelpaviricetes	Patatavirales	Potyviridae	Plants
**NC_031687**	Centovirus AC	AC	mosquito	NA	Pisoniviricetes	Picornavirales	Dicistroviridae	Invertebrates
**NC_002198**	Cereal yellow dwarf virus RPS	NA	NA	NA	Pisoniviricetes	Sobelivirales	Solemoviridae	Plants
**NC_004751**	Cereal yellow dwarf virus RPV	NY	NA	NA	Pisoniviricetes	Sobelivirales	Solemoviridae	Plants
**NC_012212**	Chaetoceros socialis forma radians RNA virus 1	NA	NA	1	Pisoniviricetes	Picornavirales	Marnaviridae	Chromista
**NC_055125**	Chaetoceros species RNA virus 02	Csp02RNAV01	Chaetoceros sp.	NA	Pisoniviricetes	Picornavirales	Marnaviridae	Chromista
**NC_038321**	Chaetoceros tenuissimus RNA virus 01	CtenRNAV01	Chaetoceros tenuissimus Meunier	NA	Pisoniviricetes	Picornavirales	Marnaviridae	Chromista
**NC_025889**	Chaetoceros tenuissimus RNA virus type II	SS10-16V	Chaetoceros tenuissimus strain 2-10	NA	Pisoniviricetes	Picornavirales	Marnaviridae	Chromista
**NC_036389**	Chequa iflavirus	A14-49.4	Cherax quadricarinatus	NA	Pisoniviricetes	Picornavirales	Iflaviridae	Invertebrates
**NC_015414**	Cherry leaf roll virus	E395	Rheum sp.	RNA 1	Pisoniviricetes	Picornavirales	Secoviridae	Plants
**NC_015415**	Cherry leaf roll virus	E395	Rheum sp.	RNA 2	Pisoniviricetes	Picornavirales	Secoviridae	Plants
**NC_006272**	Cherry rasp leaf virus	potato isolate	NA	RNA 2	Pisoniviricetes	Picornavirales	Secoviridae	Plants
**NC_006271**	Cherry rasp leaf virus	potato	NA	NA	Pisoniviricetes	Picornavirales	Secoviridae	Plants
**NC_003790**	Chicken astrovirus	G-4260	NA	NA	Stelpaviricetes	Stellavirales	Astroviridae	Vertebrates
**NC_033081**	Chicken calicivirus	RS/BR/2015	Gallus gallus	NA	Pisoniviricetes	Picornavirales	Caliciviridae	Vertebrates
**NC_039235**	Chicken megrivirus	chicken/B21-CHV/2012/HUN	Gallus gallus domesticus (domestic chicken)	NA	Pisoniviricetes	Picornavirales	Picornaviridae	Vertebrates
**NC_025432**	Chicken orivirus 1	chicken/Pf-CHK1/2013/HUN	Gallus gallus domesticus (domestic chicken)	NA	Pisoniviricetes	Picornavirales	Picornaviridae	Vertebrates
**NC_024765**	Chicken picornavirus 1	55C	Gallus gallus	NA	Pisoniviricetes	Picornavirales	Picornaviridae	Vertebrates

**NC_024766**	Chicken picornavirus 2	44C	Gallus gallus	NA	Pisoniviricetes	Picornavirales	Picornaviridae	Vertebrates
**NC_024767**	Chicken picornavirus 3	45C	Gallus gallus	NA	Pisoniviricetes	Picornavirales	Picornaviridae	Vertebrates
**NC_024768**	Chicken picornavirus 4	5C	Gallus gallus	NA	Pisoniviricetes	Picornavirales	Picornaviridae	Vertebrates
**NC_008249**	Chickpea chlorotic stunt virus	Et-fb-am1	faba bean	NA	Pisoniviricetes	Sobelivirales	Solemoviridae	Plants
**NC_016044**	Chilli ringspot virus	ChiRSV-HN/14	chilli	NA	Stelpaviricetes	Patatavirales	Potyviridae	Plants
**NC_005778**	Chilli veinal mottle virus	NA	Capsicum annuum	NA	Stelpaviricetes	Patatavirales	Potyviridae	Plants
**NC_046961**	Chinese broad-headed pond turtle arterivirus	WHWGC150683	Mauremys megalocephala	NA	Pisoniviricetes	Nidovirales	Arnidovirineae	Vertebrates
**NC_018455**	Chinese yam necrotic mosaic virus	PES3	Dioscorea opposita	NA	Stelpaviricetes	Patatavirales	Potyviridae	Plants
**NC_026812**	Chinook salmon bafinivirus	NIDO	Oncorhynchus tshawytscha	NA	Pisoniviricetes	Nidovirales	Tornidovirineae	Vertebrates
**NC_016443**	Chocolate lily virus A	KP2	Dichopogon strictus	RNA 1	Pisoniviricetes	Picornavirales	Secoviridae	Plants
**NC_016444**	Chocolate lily virus A	KP2	Dichopogon strictus	RNA 2	Pisoniviricetes	Picornavirales	Secoviridae	Plants
**NC_021564**	Citrus vein enation virus	VE-1	Citrus sp.	NA	Pisoniviricetes	Sobelivirales	Solemoviridae	Plants
**NC_003536**	Clover yellow vein virus	No.30	NA	NA	Stelpaviricetes	Patatavirales	Potyviridae	Plants
**NC_030840**	Coccinia mottle virus	Su12-25	Coccinia grandis	NA	Stelpaviricetes	Patatavirales	Potyviridae	Plants
**NC_002618**	Cocksfoot mottle virus	NA	NA	NA	Pisoniviricetes	Sobelivirales	Solemoviridae	Plants
**NC_003742**	Cocksfoot streak virus	NA	NA	NA	Stelpaviricetes	Patatavirales	Potyviridae	Plants
**NC_043077**	Cocoa necrosis virus	ATCC PV-283	NA	NA	Pisoniviricetes	Picornavirales	Secoviridae	Plants
**NC_020072**	Colombian datura virus	NA	Nicotiana tabacum	NA	Stelpaviricetes	Patatavirales	Potyviridae	Plants
**NC_034972**	Coronavirus AcCoV-JC34	AcCoV-JC34	Apodemus chevrieri	NA	Pisoniviricetes	Nidovirales	Cornidovirineae	Vertebrates
**NC_012800**	Cosavirus A	553	Homo sapiens	NA	Pisoniviricetes	Picornavirales	Picornaviridae	Vertebrates
**NC_025961**	Cosavirus JMY-2014	Cosa-CHN	Homo sapiens	NA	Pisoniviricetes	Picornavirales	Picornaviridae	Vertebrates
**NC_055568**	Costus stripe mosaic virus	BR1	Costus spiralis (spiral ginger)	NA	Stelpaviricetes	Patatavirales	Potyviridae	Plants
**NC_014545**	Cotton leafroll dwarf virus	ARG	Gossypium hirsutum (cotton)	NA	Pisoniviricetes	Sobelivirales	Solemoviridae	Plants
**NC_000940**	Cowden I virus	Cowden	NA	NA	Pisoniviricetes	Picornavirales	Caliciviridae	Vertebrates
**NC_004013**	Cowpea aphid-borne mosaic virus	CABMV-Z	NA	NA	Stelpaviricetes	Patatavirales	Potyviridae	Plants
**NC_003550**	Cowpea mosaic virus	NA	NA	NA	Pisoniviricetes	Picornavirales	Secoviridae	Plants
**NC_003549**	Cowpea mosaic virus	NA	NA	RNA 1	Pisoniviricetes	Picornavirales	Secoviridae	Plants
**NC_034246**	Cowpea polerovirus 1	BE167	Vigna unguiculata	NA	Pisoniviricetes	Sobelivirales	Solemoviridae	Plants
**NC_034247**	Cowpea polerovirus 2	BE179	Vigna unguiculata	NA	Pisoniviricetes	Sobelivirales	Solemoviridae	Plants
**NC_003545**	Cowpea severe mosaic virus	NA	NA	RNA 1	Pisoniviricetes	Picornavirales	Secoviridae	Plants
**NC_003544**	Cowpea severe mosaic virus	NA	NA	RNA 2	Pisoniviricetes	Picornavirales	Secoviridae	Plants
**NC_038307**	Coxsackievirus B3	NA	NA	NA	Pisoniviricetes	Picornavirales	Picornaviridae	Vertebrates
**NC_003924**	Cricket paralysis virus	NA	NA	NA	Pisoniviricetes	Picornavirales	Dicistroviridae	Invertebrates
**NC_025219**	Cripavirus NB-1/2011/HUN	NB-1/2011/HUN	Pipistrellus pipistrellus (bat)	NA	Pisoniviricetes	Picornavirales	Dicistroviridae	Invertebrates
**NC_025474**	Crohivirus A	ZM54	Crocidura hirta	NA	Pisoniviricetes	Picornavirales	Picornaviridae	Vertebrates
**NC_033819**	Crohivirus B	NA	Eidolon helvum	NA	Pisoniviricetes	Picornavirales	Picornaviridae	Vertebrates
**NC_006941**	Cucumber vein yellowing virus	ALM32	NA	NA	Stelpaviricetes	Patatavirales	Potyviridae	Plants
**NC_003688**	Cucurbit aphid-borne yellows virus	N	NA	NA	Pisoniviricetes	Sobelivirales	Solemoviridae	Plants
**NC_038760**	Cucurbit mild mosaic virus	Beijing	cucurbit	RNA1	Pisoniviricetes	Picornavirales	Secoviridae	Plants
**NC_038759**	Cucurbit mild mosaic virus	Beijing	curbit	RNA2	Pisoniviricetes	Picornavirales	Secoviridae	Plants
**NC_035134**	Cucurbit vein banding virus	3.1	Cucurbita maxima (Zapallito)	NA	Stelpaviricetes	Patatavirales	Potyviridae	Plants
**NC_040647**	Culex Bastrovirus-like virus	CAVL/Fresno	Culex sp. (mosquito)	NA	Stelpaviricetes	Stellavirales	Astroviridae	Unknown
**NC_040646**	Culex Iflavi-like virus 1	CIVL1/Sutter	Culex sp. (mosquito)	NA	Pisoniviricetes	Picornavirales	Iflaviridae	Invertebrates
**NC_040574**	Culex Iflavi-like virus 4	CIVL/Kern	Culex sp. (mosquito)	NA	Pisoniviricetes	Picornavirales	Iflaviridae	Invertebrates
**NC_040832**	Culex Iflavi-like virus 4	CIVL4-Sonoma	Culex sp. (mosquito)	NA	Pisoniviricetes	Picornavirales	Iflaviridae	Invertebrates
**NC_040716**	Culex Iflavi-like virus 4	CIVL4/Fresno	Culex sp. (mosquito)	NA	Pisoniviricetes	Picornavirales	Iflaviridae	Invertebrates

**NC_029038**	Currant latent virus	NA	red currant cv. Holandsky cerveny	RNA 1	Pisoniviricetes	Picornavirales	Secoviridae	Plants
**NC_029036**	Currant latent virus	NA	red currant cv. Holandsky cerveny	RNA 2	Pisoniviricetes	Picornavirales	Secoviridae	Plants
**NC_003791**	Cycas necrotic stunt virus	NA	NA	RNA 1	Pisoniviricetes	Picornavirales	Secoviridae	Plants
**NC_003792**	Cycas necrotic stunt virus	NA	NA	RNA 2	Pisoniviricetes	Picornavirales	Secoviridae	Plants
**NC_027123**	Cymbidium chlorotic mosaic virus	Cym92-20	Cymbidium goeringii (spring orchid)	NA	Pisoniviricetes	Sobelivirales	Solemoviridae	Plants
**NC_038297**	Dak Nong virus	HL30	Culex tritaeniorhynchus	NA	Pisoniviricetes	Nidovirales	Mesnidovirineae	Invertebrates
**NC_043078**	Dandelion yellow mosaic virus	DSM2	NA	NA	Pisoniviricetes	Picornavirales	Secoviridae	Plants
**NC_008028**	Daphne mosaic virus	NA	Daphne mezereum	NA	Stelpaviricetes	Patatavirales	Potyviridae	Plants
**NC_038984**	Daphne virus Y	SK	Daphne odora Thunb.	NA	Stelpaviricetes	Patatavirales	Potyviridae	Plants
**NC_003537**	Dasheen mosaic virus	M13	Zantedeschia aethiopica	NA	Stelpaviricetes	Patatavirales	Potyviridae	Plants
**NC_026509**	DeBrazza's monkey arterivirus	PREDICT-06530	Cercopithecus neglectus	NA	Pisoniviricetes	Nidovirales	Arnidovirineae	Vertebrates
**NC_004830**	Deformed wing virus	NA	Apis mellifera	NA	Pisoniviricetes	Picornavirales	Iflaviridae	Invertebrates
**NC_029131**	Delisea pulchra RNA virus	Delisea	Delisea pulchra	NA	Pisoniviricetes	Picornavirales		Chromista
**NC_055520**	Dendrobium chlorotic mosaic virus	98-De-31	Dendrobium smillieae	NA	Stelpaviricetes	Patatavirales	Potyviridae	Plants
**NC_034384**	Diamondback moth iflavirus	Guangzhou	Plutella xylostella	NA	Pisoniviricetes	Picornavirales	Iflaviridae	Invertebrates
**NC_036586**	Dianke virus	SEN235030	Mastomys natalensis	NA	Pisoniviricetes	Nidovirales	Mesnidovirineae	Invertebrates
**NC_025835**	Dinocampus coccinellae paralysis virus	Quebec2013	Dinocampus coccinellae (Schrank, 1802)	NA	Pisoniviricetes	Picornavirales	Iflaviridae	Invertebrates
**NC_055506**	Dioscorea mosaic virus	DMV-FL	Dioscorea bulbifera	NA	Stelpaviricetes	Patatavirales	Potyviridae	Plants
**NC_031766**	Dioscorea mosaic associated virus	goiana	Dioscorea rotundata	RA11	Pisoniviricetes	Picornavirales	Secoviridae	Plants
**NC_031763**	Dioscorea mosaic associated virus	goiana	Dioscorea rotundata	RA22	Pisoniviricetes	Picornavirales	Secoviridae	Plants
**NC_021197**	Donkey orchid virus A	SW3.1	Diuris corymbosa	NA	Stelpaviricetes	Patatavirales	Potyviridae	Plants
**NC_027711**	Dromedary astrovirus	DcAstV-274	Camelus dromedarius	NA	Stelpaviricetes	Stellavirales	Astroviridae	Vertebrates
**NC_038310**	Dromedary camel enterovirus 19CC	19CC	dromedary	NA	Pisoniviricetes	Picornavirales	Picornaviridae	Vertebrates
**NC_001834**	Drosophila C virus	EB	NA	NA	Pisoniviricetes	Picornavirales	Dicistroviridae	Invertebrates
**NC_012437**	Duck astrovirus C-NGB	C-NGB	duck	NA	Stelpaviricetes	Stellavirales	Astroviridae	Vertebrates
**NC_048214**	Duck coronavirus	DK/GD/27/2014	duck	NA	Pisoniviricetes	Nidovirales	Cornidovirineae	Vertebrates
**NC_008250**	Duck hepatitis A virus 1	R85952; ATCC VR-191	NA	NA	Pisoniviricetes	Picornavirales	Picornaviridae	Vertebrates
**NC_023985**	Duck aalivirus 1	GL/12	Pekin duck	NA	Pisoniviricetes	Picornavirales	Picornaviridae	Vertebrates
**NC_055483**	East Asian Passiflora distortion virus	PY-AK	Passiflora edulis	NA	Stelpaviricetes	Patatavirales	Potyviridae	Plants
**NC_007728**	East Asian Passiflora virus	AO	NA	NA	Stelpaviricetes	Patatavirales	Potyviridae	Plants
**NC_005092**	Ectropis obliqua picorna-like virus	NA	Ectropis obliqua	NA	Pisoniviricetes	Picornavirales	Iflaviridae	Invertebrates
**NC_022332**	Eel picornavirus 1	F15/05	Anguilla anguilla	NA	Pisoniviricetes	Picornavirales	Picornaviridae	Vertebrates
**NC_001479**	Encephalomyocarditis virus	Ruckert	NA	NA	Pisoniviricetes	Picornavirales	Picornaviridae	Vertebrates
**NC_034273**	Endive necrotic mosaic virus	ENMV-FR	lettuce	NA	Stelpaviricetes	Patatavirales	Potyviridae	Plants
**NC_030454**	enterovirus A114	V13-0285	Homo sapiens	NA	Pisoniviricetes	Picornavirales	Picornaviridae	Vertebrates
**NC_033695**	Enterovirus AN12	AN12	Bos taurus	NA	Pisoniviricetes	Picornavirales	Picornaviridae	Vertebrates
**NC_021220**	Enterovirus F	BEV-261; M2; RM2	NA	NA	Pisoniviricetes	Picornavirales	Picornaviridae	Vertebrates
**NC_034267**	Enterovirus goat/JL14	JL14	Capra hircus	NA	Pisoniviricetes	Picornavirales	Picornaviridae	Vertebrates
**NC_010415**	Enterovirus J	Simian enterovirus SV6	NA	NA	Pisoniviricetes	Picornavirales	Picornaviridae	Vertebrates
**NC_013695**	Enterovirus J	Simian picornavirus strain N203	NA	NA	Pisoniviricetes	Picornavirales	Picornaviridae	Vertebrates
**NC_029905**	Enterovirus SEV-gx	SEV-gx	Macaca mulatta	NA	Pisoniviricetes	Picornavirales	Picornaviridae	Vertebrates
**NC_024073**	Enterovirus sp.	CPML_8109/08	Mandrillus sphinx	NA	Pisoniviricetes	Picornavirales	Picornaviridae	Vertebrates
**NC_002532**	Equine arteritis virus	Bucyrus	NA	NA	Pisoniviricetes	Nidovirales	Arnidovirineae	Vertebrates
**NC_039209**	Equine rhinitis A virus	PERV-1	horse	NA	Pisoniviricetes	Picornavirales	Picornaviridae	Vertebrates
**NC_003983**	Equine rhinitis B virus 1	P1436 /71	horse	NA	Pisoniviricetes	Picornavirales	Picornaviridae	Vertebrates
**NC_043213**	Equine torovirus	Berne	NA	NA	Pisoniviricetes	Nidovirales	Tornidovirineae	Vertebrates
**NC_031339**	Euphorbia ringspot virus	PV-0902	Euphorbia milii x lophogona	NA	Stelpaviricetes	Patatavirales	Potyviridae	Plants

**NC_002615**	European brown hare syndrome virus	EBHSV-GD	NA	NA	Pisoniviricetes	Picornavirales	Caliciviridae	Vertebrates
**NC_032087**	Euscelidius variegatus virus 1	to-1	Euscelidius variegatus	NA	Pisoniviricetes	Picornavirales	Iflaviridae	Invertebrates
**NC_055495**	Faba bean polerovirus 1	5253	faba bean	NA	Pisoniviricetes	Sobelivirales	Solemoviridae	Plants
**NC_035779**	Falcon picornavirus	falcon/HA18-080/2014/HUN	Falco vespertinus	NA	Pisoniviricetes	Picornavirales	Picornaviridae	Vertebrates
**NC_026921**	Falcovirus A1	kestrel/VOVE0622/2013/HUN	Falco tinnunculus (common kestrel)	NA	Pisoniviricetes	Picornavirales	Picornaviridae	Vertebrates
**NC_035675**	Fathead minnow calicivirus	FHMCV/USA/MN/2012	Pimephales promelas	NA	Pisoniviricetes	Picornavirales	Caliciviridae	Vertebrates
**NC_038295**	Fathead minnow nidovirus	NA	Pimephales promelas (fathead minnow)	NA	Pisoniviricetes	Nidovirales	Tornidovirineae	Vertebrates
**NC_039212**	Fathead minnow picornavirus	FHMPV-1	Pimephales promelas	NA	Pisoniviricetes	Picornavirales	Picornaviridae	Vertebrates
**NC_022249**	Feline astrovirus 2	1637F	Felis catus	NA	Stelpaviricetes	Stellavirales	Astroviridae	Vertebrates
**NC_024701**	Feline astrovirus D1	FAstV-D1	Felis catus	NA	Stelpaviricetes	Stellavirales	Astroviridae	Vertebrates
**NC_001481**	Feline calicivirus	Urbana	NA	NA	Pisoniviricetes	Picornavirales	Caliciviridae	Vertebrates
**NC_002306**	Feline infectious peritonitis virus	79-1146	NA	NA	Pisoniviricetes	Nidovirales	Cornidovirineae	Vertebrates
**NC_016156**	Feline picornavirus	073F	Felis catus	NA	Pisoniviricetes	Picornavirales	Picornaviridae	Vertebrates
**NC_022802**	Feline sakobuvirus A	FFUP1	Felis catus	NA	Pisoniviricetes	Picornavirales	Picornaviridae	Vertebrates
**NC_030292**	Ferret coronavirus	FRCoV-NL-2010	Mustela putorius (ferret)	NA	Pisoniviricetes	Nidovirales	Cornidovirineae	Vertebrates
**NC_034453**	Ferret parechovirus	MpPeV1	Mustela putorius furo	NA	Pisoniviricetes	Picornavirales	Picornaviridae	Vertebrates
**NC_039210**	Foot-and-mouth disease virus O	o6pirbright iso58	NA	NA	Pisoniviricetes	Picornavirales	Picornaviridae	Vertebrates
**NC_026439**	African pouched rat arterivirus	PREDICT-06509	Cricetomys emini	NA	Pisoniviricetes	Nidovirales	Arnidovirineae	Vertebrates
**NC_023021**	Formica exsecta virus 1	Fex1	Formica exsecta	NA	Pisoniviricetes	Picornavirales	Dicistroviridae	Invertebrates
**NC_023022**	Formica exsecta virus 2	Fex2	Formica exsecta	NA	Pisoniviricetes	Picornavirales	Iflaviridae	Invertebrates
**NC_014064**	Freesia mosaic virus	NA	NA	NA	Stelpaviricetes	Patatavirales	Potyviridae	Plants
**NC_010954**	Fritillary virus Y	Pan'an	Fritillaria thunbergii	NA	Stelpaviricetes	Patatavirales	Potyviridae	Plants
**NC_030870**	Fusarium poae mycovirus 2	NA	Fusarium poae MAFF 240374	NA	Yadokarivirales	Yadokariviridae	Betayadokarivirus	Fungi
**NC_024770**	gallivirus A1	518C	Gallus gallus	NA	Pisoniviricetes	Picornavirales	Picornaviridae	Vertebrates
**NC_018400**	gallivirus A1	turkey/M176/2011/HUN	Meleagris gallopavo (domestic turkey)	NA	Pisoniviricetes	Picornavirales	Picornaviridae	Vertebrates
**NC_028964**	Gallivirus Pf-CHK1/GV	Pf-CHK1/GV	Gallus gallus domesticus (domestic chicken)	NA	Pisoniviricetes	Picornavirales	Picornaviridae	Vertebrates
**NC_010306**	Gill-associated virus	NA	Penaeus monodon	NA	Pisoniviricetes	Nidovirales	Ronidovirineae	Invertebrates
**NC_038562**	Gloriosa stripe mosaic virus	CB	Sandersonia aurantiaca (Christmas bell)	NA	Stelpaviricetes	Patatavirales	Potyviridae	Plants
**NC_034976**	Goat torovirus	SZ	Capra hircus	NA	Pisoniviricetes	Nidovirales	Tornidovirineae	Vertebrates
**NC_055470**	Gomphocarpus mosaic virus	NA	Gomphocarpus physocarpus	1	Stelpaviricetes	Patatavirales	Potyviridae	Plants
**NC_034567**	Goose astrovirus	FLX	Hortobagy goose	NA	Stelpaviricetes	Stellavirales	Astroviridae	Vertebrates
**NC_024078**	Goose calicivirus	N	goose	NA	Pisoniviricetes	Picornavirales	Caliciviridae	Vertebrates
**NC_029052**	Goose dicistrovirus	UW1	goose	NA	Pisoniviricetes	Picornavirales	Dicistroviridae	Invertebrates
**NC_040684**	Goose picornavirus 1	goose/NLSZK2/HUN/2013	Anser albifrons (greater white-fronted goose)	NA	Pisoniviricetes	Picornavirales	Picornaviridae	Vertebrates
**NC_026733**	Graminella nigrifrons virus 1	Ohio	Graminella nigrifrons	NA	Pisoniviricetes	Picornavirales	Iflaviridae	Invertebrates
**NC_018383**	Grapevine Anatolian ringspot virus	A34	Vitis vinifera	RNA 1	Pisoniviricetes	Picornavirales	Secoviridae	Plants
**NC_018384**	Grapevine Anatolian ringspot virus	NA	NA	RNA 2	Pisoniviricetes	Picornavirales	Secoviridae	Plants
**NC_015492**	Grapevine Bulgarian latent virus	Serb1	Vitis vinifera	1	Pisoniviricetes	Picornavirales	Secoviridae	Plants
**NC_015493**	Grapevine Bulgarian latent virus	Serb1	Vitis vinifera	2	Pisoniviricetes	Picornavirales	Secoviridae	Plants
**NC_003622**	Grapevine chrome mosaic virus	NA	NA	RNA 1	Pisoniviricetes	Picornavirales	Secoviridae	Plants
**NC_003621**	Grapevine chrome mosaic virus	NA	NA	RNA 2	Pisoniviricetes	Picornavirales	Secoviridae	Plants
**NC_017939**	Grapevine deformation virus	N66	Vitis vinifera, cultivar Dimrit	RNA 1	Pisoniviricetes	Picornavirales	Secoviridae	Plants
**NC_017938**	Grapevine deformation virus	NA	NA	RNA 2	Pisoniviricetes	Picornavirales	Secoviridae	Plants
**NC_034836**	Grapevine enamovirus 1	SE-BR	Vitis vinifera cv. Semillon	NA	Pisoniviricetes	Sobelivirales	Solemoviridae	Plants
**NC_039072**	Grapevine fabavirus	BB	Vitis vinifera cv. Black Beet	RNA2	Pisoniviricetes	Picornavirales	Secoviridae	Plants

**NC_039073**	Grapevine fabavirus	NP	Vitis vinifera cv. Nagano Purple	RNA1	Pisoniviricetes	Picornavirales	Secoviridae	Plants
**NC_003623**	Grapevine fanleaf virus	F13	NA	NA	Pisoniviricetes	Picornavirales	Secoviridae	Plants
**NC_003615**	Grapevine fanleaf virus	F13	NA	RNA 1	Pisoniviricetes	Picornavirales	Secoviridae	Plants
**NC_038509**	Groundnut rosette assistor virus	NA	Arachis hypogaea	NA	Pisoniviricetes	Sobelivirales	Solemoviridae	Plants
**NC_046959**	Guangdong greater green snake arterivirus	LPSG2430	Cyclophiops major	NA	Pisoniviricetes	Nidovirales	Arnidovirineae	Vertebrates
**NC_046963**	Guangdong red-banded snake torovirus	LPSF30546	Lycodon rufozonatus	NA	Pisoniviricetes	Nidovirales	Tornidovirineae	Vertebrates
**NC_021786**	Habenaria mosaic virus	Ha-1	Habenaria radiata (terrestrial orchid)	NA	Stelpaviricetes	Patatavirales	Potyviridae	Plants
**NC_043544**	rafivirus B1	LPXYC222841	Gekko similignum	NA	Pisoniviricetes	Picornavirales	Picornaviridae	Vertebrates
**NC_046958**	Hainan oligodon formosanus arterivirus	LPSF32245	Oligodon formosanus	NA	Pisoniviricetes	Nidovirales	Arnidovirineae	Vertebrates
**NC_022611**	Halyomorpha halys virus	Beltsville	Halyomorpha halys	NA	Pisoniviricetes	Picornavirales	Iflaviridae	Invertebrates
**NC_020899**	Alphamesonivirus 2	A4/CI/2004	Culex sp.	NA	Pisoniviricetes	Nidovirales	Mesnidovirineae	Invertebrates
**NC_015394**	Hardenbergia mosaic virus	HarMV-57.2	Hardenbergia comptoniana	NA	Stelpaviricetes	Patatavirales	Potyviridae	Plants
**NC_034617**	Harrier picornavirus 1	harrier/MR-01/HUN/2014	Circus aeruginosus (Western Marsh-harrier)	NA	Pisoniviricetes	Picornavirales	Picornaviridae	Vertebrates
**NC_040611**	Hedgehog dicipivirus	hedgehog/H14/2015/HUN	Erinaceus roumanicus (Northern white-breasted hedgehog)	NA	Pisoniviricetes	Picornavirales	Picornaviridae	Vertebrates
**NC_028365**	Hedgehog hepatovirus Igel8Erieur2014	Igel8Erieur2014	Erinaceus europaeus	NA	Pisoniviricetes	Picornavirales	Picornaviridae	Vertebrates
**NC_024016**	Heliconius erato iflavirus	HeratoCRSEC	Heliconius erato	NA	Pisoniviricetes	Picornavirales	Iflaviridae	Invertebrates
**NC_033619**	Helicoverpa armigera iflavirus	HBLF2013-1	Helicoverpa armigera (cotton bollworm)	NA	Pisoniviricetes	Picornavirales	Iflaviridae	Invertebrates
**NC_001489**	Hepatovirus A	NA	NA	NA	Pisoniviricetes	Picornavirales	Picornaviridae	Vertebrates
**NC_028366**	hepatovirus H2	M32Eidhel2010	Eidolon helvum	NA	Pisoniviricetes	Picornavirales	Picornaviridae	Vertebrates
**NC_007518**	Heterocapsa circularisquama RNA virus 01	HcRNAV34	NA	NA	Pisoniviricetes	Sobelivirales	Alvernaviridae	Chromista
**NC_005281**	Heterosigma akashiwo RNA virus	HaRNAV-SOG263	NA	NA	Pisoniviricetes	Picornavirales	Marnaviridae	Chromista
**NC_003782**	Himetobi P virus	NA	Laodelphax striatellus	NA	Pisoniviricetes	Picornavirales	Dicistroviridae	Invertebrates
**NC_017967**	Hippeastrum mosaic virus	Marijiniup 1	Hippeastrum sp.	NA	Stelpaviricetes	Patatavirales	Potyviridae	Plants
**NC_013443**	HMO Astrovirus A	NI-295	Homo sapiens	NA	Stelpaviricetes	Stellavirales	Astroviridae	Vertebrates
**NC_034444**	Hom-1 vesivirus	Hom-1	NA	NA	Pisoniviricetes	Picornavirales	Caliciviridae	Vertebrates
**NC_008029**	Homalodisca coagulata virus 1	NA	NA	NA	Pisoniviricetes	Picornavirales	Dicistroviridae	Invertebrates
**NC_005904**	Hordeum mosaic virus	ATCC PV81	NA	NA	Stelpaviricetes	Patatavirales	Potyviridae	Plants
**NC_033152**	Hubei picorna-like virus 81	QTM27117	Odonata	NA	Pisoniviricetes	Picornavirales	Polycipiviridae	Invertebrates
**NC_032222**	Hubei picorna-like virus 82	spider133992	spiders	NA	Pisoniviricetes	Picornavirales	Polycipiviridae	Invertebrates
**NC_032912**	Hubei Poty-like virus 1	SCM51506	Diptera	NA	Stelpaviricetes	Patatavirales	Potyviridae	Plants
**NC_024472**	Human astrovirus BF34	BF34	Homo sapiens	NA	Stelpaviricetes	Stellavirales	Astroviridae	Vertebrates
**NC_001943**	Human astrovirus	NA	NA	NA	Stelpaviricetes	Stellavirales	Astroviridae	Vertebrates
**NC_002645**	Human coronavirus 229E	NA	NA	NA	Pisoniviricetes	Nidovirales	Cornidovirineae	Vertebrates
**NC_005831**	Human coronavirus NL63	Amsterdam I	NA	NA	Pisoniviricetes	Nidovirales	Cornidovirineae	Vertebrates
**NC_006213**	Human coronavirus OC43	ATCC VR-759	NA	NA	Pisoniviricetes	Nidovirales	Cornidovirineae	Vertebrates
**NC_012801**	Human cosavirus B	2263	Homo sapiens	NA	Pisoniviricetes	Picornavirales	Picornaviridae	Vertebrates
**NC_012802**	Cosavirus D	5004	Homo sapiens	NA	Pisoniviricetes	Picornavirales	Picornaviridae	Vertebrates
**NC_012798**	Cosavirus E	HCoSV-E1	Homo sapiens	NA	Pisoniviricetes	Picornavirales	Picornaviridae	Vertebrates
**NC_034385**	Cosavirus F	PK5006	Homo sapiens	NA	Pisoniviricetes	Picornavirales	Picornaviridae	Vertebrates
**NC_023984**	Human cosavirus	Cosavirus_Amsterdam_1994	Homo sapiens	NA	Pisoniviricetes	Picornavirales	Picornaviridae	Vertebrates
**NC_038306**	Coxsackievirus A2	CA2	NA	NA	Pisoniviricetes	Picornavirales	Picornaviridae	Vertebrates
**NC_038308**	enterovirus D68	Fermon	NA	NA	Pisoniviricetes	Picornavirales	Picornaviridae	Vertebrates
**NC_001612**	Enterovirus A	NA	NA	NA	Pisoniviricetes	Picornavirales	Picornaviridae	Vertebrates
**NC_001472**	Enterovirus B	NA	NA	NA	Pisoniviricetes	Picornavirales	Picornaviridae	Vertebrates
**NC_001430**	Enterovirus D	Enterovirus 70	NA	NA	Pisoniviricetes	Picornavirales	Picornaviridae	Vertebrates
**NC_001897**	Parechovirus A	Gregory	NA	NA	Pisoniviricetes	Picornavirales	Picornaviridae	Vertebrates
**NC_038311**	rhinovirus A1	ATCC VR-1559	NA	NA	Pisoniviricetes	Picornavirales	Picornaviridae	Vertebrates
**NC_038312**	rhinovirus B3	NA	NA	NA	Pisoniviricetes	Picornavirales	Picornaviridae	Vertebrates
**NC_001617**	Rhinovirus A	NA	NA	NA	Pisoniviricetes	Picornavirales	Picornaviridae	Vertebrates

**NC_009996**	Rhinovirus C	24	NA	NA	Pisoniviricetes	Picornavirales	Picornaviridae	Vertebrates
**NC_038878**	Human rhinovirus NAT001	NAT001	NA	NA	Pisoniviricetes	Picornavirales	Picornaviridae	Vertebrates
**NC_010810**	Human TMEV-like cardiovirus	NA	NA	NA	Pisoniviricetes	Picornavirales	Picornaviridae	Vertebrates
**NC_037051**	Hyacinth mosaic virus	Nannup BC28	Hyacinthus orientalis	NA	Stelpaviricetes	Patatavirales	Potyviridae	Plants
**NC_030236**	Impatiens flower break virus	Asan	Impatiens walleriana hook.	NA	Stelpaviricetes	Patatavirales	Potyviridae	Plants
**NC_011536**	Imperata yellow mottle virus	NA	Imperata cylindrica	NA	Pisoniviricetes	Sobelivirales	Solemoviridae	Plants
**NC_048213**	Infectious bronchitis virus	Ind-TN92-03	chicken	NA	Pisoniviricetes	Nidovirales	Cornidovirineae	Vertebrates
**NC_003781**	Infectious flacherie virus	NA	NA	NA	Pisoniviricetes	Picornavirales	Iflaviridae	Invertebrates
**NC_018833**	Iranian johnsongrass mosaic virus	Shz	Johnson grass	NA	Stelpaviricetes	Patatavirales	Potyviridae	Plants
**NC_029076**	Iris severe mosaic virus	BJ	iris	NA	Stelpaviricetes	Patatavirales	Potyviridae	Plants
**NC_009025**	Israeli acute paralysis virus	NA	NA	NA	Pisoniviricetes	Picornavirales	Dicistroviridae	Invertebrates
**NC_000947**	Japanese yam mosaic virus	NA	NA	NA	Stelpaviricetes	Patatavirales	Potyviridae	Plants
**NC_029051**	Jasmine virus T	NA	jasmine	NA	Stelpaviricetes	Patatavirales	Potyviridae	Plants
**NC_003606**	Johnsongrass mosaic virus	NA	NA	NA	Stelpaviricetes	Patatavirales	Potyviridae	Plants
**NC_043488**	Kadiweu virus	BrMS-MQ10	Culex (Culex) sp.	NA	Pisoniviricetes	Nidovirales	Mesnidovirineae	Invertebrates
**NC_029053**	Kafue kinda chacma baboon virus	KKCBV-1	Chlorocebus pygerythrus	NA	Pisoniviricetes	Nidovirales	Arnidovirineae	Vertebrates
**NC_038296**	Karang Sari virus	JKT10701	Culex vishnui	NA	Pisoniviricetes	Nidovirales	Mesnidovirineae	Invertebrates
**NC_004807**	Kashmir bee virus	NA	NA	NA	Pisoniviricetes	Picornavirales	Dicistroviridae	Invertebrates
**NC_016159**	Keunjorong mosaic virus	Cheongwon	Cynanchum wilfordii	NA	Stelpaviricetes	Patatavirales	Potyviridae	Plants
**NC_033553**	Kibale red colobus virus 1	SHFV-krc1_RC61	Piliocolobus tephrosceles (red colobus monkey)	NA	Pisoniviricetes	Nidovirales	Arnidovirineae	Vertebrates
**NC_034455**	Kibale red colobus virus 2	SHFV-krc2_RC61	Piliocolobus tephrosceles (red colobus monkey)	NA	Pisoniviricetes	Nidovirales	Arnidovirineae	Vertebrates
**NC_038292**	Kibale red-tailed guenon virus 1	krtg05	red-tailed guenon	NA	Pisoniviricetes	Nidovirales	Arnidovirineae	Vertebrates
**NC_027126**	Kilifi Virus	NA	large pool of wild-caught Drosophila	NA	Pisoniviricetes	Picornavirales		Invertebrates
**NC_031749**	King virus	UWV2	Yuma myotis	NA	Pisoniviricetes	Picornavirales	Iflaviridae	Unknown
**NC_027919**	Kobuvirus cattle/Kagoshima-1-22-KoV/2014/JPN	Kagoshima-1-22-KoV/2014/JPN	Bos taurus	NA	Pisoniviricetes	Picornavirales	Picornaviridae	Vertebrates
**NC_027918**	Kobuvirus cattle/Kagoshima-2-24-KoV/2015/JPN	Kagoshima-2-24-KoV/2015/JPN	Bos taurus	NA	Pisoniviricetes	Picornavirales	Picornaviridae	Vertebrates
**NC_007913**	Konjac mosaic virus	KoMV-F	Amorphophallus konjac K. Koch (Konjak)	NA	Stelpaviricetes	Patatavirales	Potyviridae	Plants
**NC_038317**	Kunsagivirus A	roller/SZAL6-KuV/2011/HUN	Coracias garrulus (European roller)	NA	Pisoniviricetes	Picornavirales	Picornaviridae	Vertebrates
**NC_033818**	Kunsagivirus B	NA	Eidolon helvum	NA	Pisoniviricetes	Picornavirales	Picornaviridae	Vertebrates
**NC_027128**	La Jolla virus	MAT03	large pool of wild-caught Drosophila	NA	Pisoniviricetes	Picornavirales	Iflaviridae	Invertebrates
**NC_001639**	Lactate dehydrogenase-elevating virus	Plagemann	NA	NA	Pisoniviricetes	Nidovirales	Arnidovirineae	Vertebrates
**NC_023016**	Lamium mild mosaic virus	DSMZ PV-0454	Lamium album	RNA 1	Pisoniviricetes	Picornavirales	Secoviridae	Plants
**NC_023017**	Lamium mild mosaic virus	DSMZ PV-0454	Lamium album	RNA 2	Pisoniviricetes	Picornavirales	Secoviridae	Plants
**NC_023627**	Laodelphax striatella honeydew virus 1	Nanjing	Laodelphax striatella	NA	Pisoniviricetes	Picornavirales	Iflaviridae	Invertebrates
**NC_025788**	Laodelphax striatellus picorna-like virus 2	LsPV2	Laodelphax striatella	NA	Pisoniviricetes	Picornavirales	Iflaviridae	Invertebrates
**NC_035450**	Lasius neglectus virus 1	Cambridge-Lne	Lasius neglectus (ant)	NA	Pisoniviricetes	Picornavirales	Polycipiviridae	Invertebrates
**NC_035456**	Lasius niger virus 1	Cambridge-Lni	Lasius niger (ant)	NA	Pisoniviricetes	Picornavirales	Polycipiviridae	Invertebrates
**NC_004011**	Leek yellow stripe virus	Yuhang GYH	garlic	NA	Stelpaviricetes	Patatavirales	Potyviridae	Plants
**NC_043487**	Lelystad virus	NA	NA	NA	Pisoniviricetes	Nidovirales	Arnidovirineae	Vertebrates
**NC_026315**	Lesavirus 1	Mis101308/2012	Lemur catta	NA	Pisoniviricetes	Picornavirales	Picornaviridae	Vertebrates
**NC_026316**	Lesavirus 2	Nai108015/2012	Varecia variegata	NA	Pisoniviricetes	Picornavirales	Picornaviridae	Vertebrates
**NC_027706**	Lettuce Italian necrotic virus	I234	Lactuca sativa cv. Romana	NA	Stelpaviricetes	Patatavirales	Potyviridae	Plants
**NC_003605**	Lettuce mosaic virus	E	NA	NA	Stelpaviricetes	Patatavirales	Potyviridae	Plants
**NC_035214**	Lettuce necrotic leaf curl virus	5317015	Lactuca sativa	RNA 1	Pisoniviricetes	Picornavirales	Secoviridae	Plants
**NC_035219**	Lettuce necrotic leaf curl virus	5317015	Lactuca sativa	RNA 2	Pisoniviricetes	Picornavirales	Secoviridae	Plants

**NC_005288**	Lily mottle virus	Sb	Lilium sp. cv. Sorbonne	NA	Stelpaviricetes	Patatavirales	Potyviridae	Plants
**NC_040802**	Lily yellow mosaic virus	lily-bua	lily	NA	Stelpaviricetes	Patatavirales	Potyviridae	Plants
**NC_032126**	livupivirus A1	newt/II-5-Pilis/2014/HUN	Lissotriton vulgaris (smooth newt)	NA	Pisoniviricetes	Picornavirales	Picornaviridae	Vertebrates
**NC_003976**	Ljungan virus	87-012	NA	NA	Pisoniviricetes	Picornavirales	Picornaviridae	Vertebrates
**NC_034835**	Longan witches broom-associated virus	Han1	longan	NA	Stelpaviricetes	Patatavirales	Potyviridae	Plants
**NC_001696**	Lucerne transient streak virus	LTSV-Can	Medicago sativa	NA	Pisoniviricetes	Sobelivirales	Solemoviridae	Plants
**NC_032730**	Lucheng Rn rat coronavirus	Lucheng-19	Rattus norvegicus	NA	Pisoniviricetes	Nidovirales	Cornidovirineae	Vertebrates
**NC_027703**	Luffa aphid-borne yellows virus	TH24	Luffa acutangula	NA	Pisoniviricetes	Sobelivirales	Solemoviridae	Plants
**NC_014898**	Lupinus mosaic virus	LU2	Lupinus polyphyllus	NA	Stelpaviricetes	Patatavirales	Potyviridae	Plants
**NC_038301**	Lygus lineolaris virus 1	LlV-1	Lygus lineolaris	NA	Pisoniviricetes	Picornavirales	Iflaviridae	Invertebrates
**NC_024497**	Lymantria dispar iflavirus 1	Ames	NA	NA	Pisoniviricetes	Picornavirales	Iflaviridae	Invertebrates
**NC_018570**	Macrobrachium rosenbergii Taihu virus	cn-taihu100401	Macrobrachium rosenbergii	NA	Pisoniviricetes	Picornavirales	Dicistroviridae	Invertebrates
**NC_016993**	Magpie-robin coronavirus HKU18	HKU18-chu3	magpie-robin	NA	Pisoniviricetes	Nidovirales	Cornidovirineae	Vertebrates
**NC_003626**	Maize chlorotic dwarf virus	Tennessee (TN)	NA	NA	Pisoniviricetes	Picornavirales	Secoviridae	Plants
**NC_003377**	Maize dwarf mosaic virus	Bulgaria	NA	NA	Stelpaviricetes	Patatavirales	Potyviridae	Plants
**NC_021484**	Maize yellow dwarf virus RMV	RMV MTFE87	Triticum aestivum	NA	Pisoniviricetes	Sobelivirales	Solemoviridae	Plants
**NC_029990**	Maize yellow dwarf virus-RMV2	NA	maize	NA	Pisoniviricetes	Sobelivirales	Solemoviridae	Plants
**NC_030922**	Mamastrovirus 1	V1347	NA	NA	Stelpaviricetes	Stellavirales	Astroviridae	Vertebrates
**NC_043102**	Mamastrovirus 18	AFCD337	Miniopterus pusillus	NA	Stelpaviricetes	Stellavirales	Astroviridae	Vertebrates
**NC_034974**	Mamastrovirus 2	K321	pig	NA	Stelpaviricetes	Stellavirales	Astroviridae	Vertebrates
**NC_025379**	Mamastrovirus 3	PAstV-GX1	swine	NA	Stelpaviricetes	Stellavirales	Astroviridae	Vertebrates
**NC_043541**	Marine RNA virus BC-1	NA	NA	NA	Pisoniviricetes	Picornavirales	Marnaviridae	Chromista
**NC_043542**	Marine RNA virus BC-2	NA	NA	NA	Pisoniviricetes	Picornavirales	Marnaviridae	Chromista
**NC_043543**	Marine RNA virus BC-3	NA	NA	NA	Pisoniviricetes	Picornavirales	Marnaviridae	Chromista
**NC_043682**	Marine RNA virus BC-4	NA	NA	NA	Pisoniviricetes	Picornavirales	Marnaviridae	Chromista
**NC_009757**	Marine RNA virus JP-A	NA	NA	NA	Pisoniviricetes	Picornavirales	Marnaviridae	Chromista
**NC_009758**	Marine RNA virus JP-B	NA	NA	NA	Pisoniviricetes	Picornavirales	Marnaviridae	Chromista
**NC_029306**	Marine RNA virus PAL128	NA	NA	NA	Pisoniviricetes	Picornavirales	Marnaviridae	Chromista
**NC_029307**	Marine RNA virus PAL156	NA	NA	NA	Pisoniviricetes	Picornavirales	Marnaviridae	Chromista
**NC_029309**	Marine RNA virus PAL473	NA	NA	NA	Pisoniviricetes	Picornavirales	Marnaviridae	Chromista
**NC_043515**	Marine RNA virus SF-1	NA	NA	NA	Pisoniviricetes	Picornavirales	Marnaviridae	Chromista
**NC_043518**	Marine RNA virus SF-2	NA	NA	NA	Pisoniviricetes	Picornavirales	Marnaviridae	Chromista
**NC_043519**	Marine RNA virus SF-3	SF-3	NA	NA	Pisoniviricetes	Picornavirales	Marnaviridae	Chromista
**NC_040674**	Marmot norovirus	HT16	Marmota himalayana	NA	Pisoniviricetes	Picornavirales	Caliciviridae	Vertebrates
**NC_040605**	Marmot sapelovirus 1	HT5	Marmota himalayana	NA	Pisoniviricetes	Picornavirales	Picornaviridae	Vertebrates
**NC_055496**	Mashua virus Y	Cam	Tropaeolum tuberosum	NA	Stelpaviricetes	Patatavirales	Potyviridae	Plants
**NC_043536**	Mediterranean ruda virus	ParP17	NA	NA	Stelpaviricetes	Patatavirales	Potyviridae	Plants
**NC_024120**	megrivirus A2	LY	Anas platyrhynchos	NA	Pisoniviricetes	Picornavirales	Picornaviridae	Vertebrates
**NC_033793**	megrivirus B3CP-APO	HN56	goose	NA	Pisoniviricetes	Picornavirales	Picornaviridae	Vertebrates
**NC_024769**	megrivirus C2	27C	Gallus gallus	NA	Pisoniviricetes	Picornavirales	Picornaviridae	Vertebrates
**NC_023858**	Melegrivirus A	turkey/B407-THV/2011/HUN	Meleagris gallopavo (domestic turkey)	NA	Pisoniviricetes	Picornavirales	Picornaviridae	Vertebrates
**NC_010809**	Melon aphid-borne yellows virus	NA	Benincasa hispida	NA	Pisoniviricetes	Sobelivirales	Solemoviridae	Plants
**NC_038765**	Melon mild mottle virus	NA	Cucumis melo	RNA 1	Pisoniviricetes	Picornavirales	Secoviridae	Plants
**NC_038766**	Melon mild mottle virus	NA	Cucumis melo	RNA 2	Pisoniviricetes	Picornavirales	Secoviridae	Plants
**NC_020900**	Alphamesonivirus 3	E9/CI/2004	Uranotaenia chorleyi	NA	Pisoniviricetes	Nidovirales	Mesnidovirineae	Invertebrates
**NC_019843**	Middle East respiratory	HCoV-EMC/2012	Homo sapiens	NA	Pisoniviricetes	Nidovirales	Cornidovirineae	Vertebrates

	syndrome-related coronavirus							
**NC_011190**	Mikania micrantha mosaic virus	GZ1	NA	RNA 1	Pisoniviricetes	Picornavirales	Secoviridae	Plants
**NC_011189**	Mikania micrantha mosaic virus	GZ1	NA	RNA 2	Pisoniviricetes	Picornavirales	Secoviridae	Plants
**NC_025112**	Mikumi yellow baboon virus 1	MYBV_M58	Papio cynocephalus	NA	Pisoniviricetes	Nidovirales	Arnidovirineae	Vertebrates
**NC_010438**	Miniopterus bat coronavirus HKU8	AFCD77	NA	NA	Pisoniviricetes	Nidovirales	Cornidovirineae	Vertebrates
**NC_034381**	Miniopterus schreibersii picornavirus 1	NA	Miniopterus schreibersii	NA	Pisoniviricetes	Picornavirales	Picornaviridae	Vertebrates
**NC_004579**	Mamastrovirus 10	NA	NA	NA	Stelpaviricetes	Stellavirales	Astroviridae	Vertebrates
**NC_019712**	Mink calicivirus	MCV-DL/2007/CN	mink	NA	Pisoniviricetes	Picornavirales	Caliciviridae	Vertebrates
**NC_023760**	Mink coronavirus strain WD1127	WD1127	Mustela vison	NA	Pisoniviricetes	Nidovirales	Cornidovirineae	Vertebrates
**NC_031338**	Moku virus	Big Island	Vespula pensylvanica	NA	Pisoniviricetes	Picornavirales	Iflaviridae	Invertebrates
**NC_035465**	Morelia viridis nidovirus	S14-1323_MVNV	Morelia viridis	NA	Pisoniviricetes	Nidovirales	Tornidovirineae	Vertebrates
**NC_009995**	Moroccan watermelon mosaic virus	TN05-76	zucchini	NA	Stelpaviricetes	Patatavirales	Potyviridae	Plants
**NC_023987**	Mosavirus A2	SZAL6-MoV/2011/HUN	Coracias garrulus (European roller)	NA	Pisoniviricetes	Picornavirales	Picornaviridae	Vertebrates
**NC_031688**	Mosquito dicistrovirus	FH1	mosquito	NA	Pisoniviricetes	Picornavirales	Dicistroviridae	Invertebrates
**NC_035218**	Motherwort yellow mottle virus	AD01	Motherwort	RNA 1	Pisoniviricetes	Picornavirales	Secoviridae	Plants
**NC_035220**	Motherwort yellow mottle virus	AD01	Motherwort	RNA 2	Pisoniviricetes	Picornavirales	Secoviridae	Plants
**NC_015935**	Mouse astrovirus M-52/USA/2008	M-52	Mus musculus	NA	Stelpaviricetes	Stellavirales	Astroviridae	Vertebrates
**NC_015936**	Mouse kobuvirus M-5/USA/2010	M-5	Peromyscus crinitus	NA	Pisoniviricetes	Picornavirales	Picornaviridae	Vertebrates
**NC_038318**	Mouse Mosavirus	Mosa.M-7	Peromyscus crinitus	NA	Pisoniviricetes	Picornavirales	Picornaviridae	Vertebrates
**NC_014793**	Mud crab virus	NA	Scylla serrata (mud crab)	NA	Pisoniviricetes	Picornavirales	Dicistroviridae	Invertebrates
**NC_038767**	Mulberry mosaic leaf roll associated virus	zj	Morus alba L.	RNA1	Pisoniviricetes	Picornavirales	Secoviridae	Plants
**NC_038768**	Mulberry mosaic leaf roll associated virus	zj	Morus alba L.	RNA2	Pisoniviricetes	Picornavirales	Secoviridae	Plants
**NC_011550**	Munia coronavirus HKU13-3514	NA	white-rumped munia	NA	Pisoniviricetes	Nidovirales	Cornidovirineae	Vertebrates
**NC_018702**	Murine astrovirus	STL 1	NA	NA	Stelpaviricetes	Stellavirales	Astroviridae	Vertebrates
**NC_048217**	Murine hepatitis virus	A59	NA	NA	Pisoniviricetes	Nidovirales	Cornidovirineae	Vertebrates
**AC_000192**	Murine hepatitis virus strain JHM	NA	NA	NA	Pisoniviricetes	Nidovirales	Cornidovirineae	Vertebrates
**NC_001846**	Murine hepatitis virus	MHV-A59	NA	NA	Pisoniviricetes	Nidovirales	Cornidovirineae	Vertebrates
**NC_001633**	Mushroom bacilliform virus	NA	NA	NA	Pisoniviricetes	Sobelivirales	Barnaviridae	Fungi
**NC_035457**	Myrmica scabrinodis virus 1	Cambridge-Msc	Myrmica scabrinodis (ant)	NA	Pisoniviricetes	Picornavirales	Polycipiviridae	Invertebrates
**NC_015874**	Nam Dinh virus	02VN178	mosquito	NA	Pisoniviricetes	Nidovirales	Mesnidovirineae	Invertebrates
**NC_046960**	Nanhai ghost shark arterivirus	NHYJS6157	Chimaera sp.	NA	Pisoniviricetes	Nidovirales	Nanidovirineae	Vertebrates
**NC_008824**	Narcissus degeneration virus	Zhangzhou	Narcissus tazetta (Chinese narcissus)	NA	Stelpaviricetes	Patatavirales	Potyviridae	Plants
**NC_023628**	Narcissus late season yellows virus	Marijiniup8	Narcissus sp.	NA	Stelpaviricetes	Patatavirales	Potyviridae	Plants
**NC_011541**	Narcissus yellow stripe virus	Zhangzhou	Narcissus tazetta var. chinensis	NA	Stelpaviricetes	Patatavirales	Potyviridae	Plants
**NC_004064**	Nebraska virus	NB	NA	NA	Pisoniviricetes	Picornavirales	Caliciviridae	Vertebrates
**NC_040798**	Nephila clavipes virus 1	SC	Nephila clavipes	NA	Pisoniviricetes	Picornavirales		Invertebrates
**NC_040542**	Nephila clavipes virus 2	SC	Nephila clavipes	NA	Pisoniviricetes	Picornavirales		Invertebrates
**NC_040675**	Nesidiocoris tenuis iflavirus 1	SD	NA	NA	Pisoniviricetes	Picornavirales	Iflaviridae	Invertebrates
**NC_007916**	Newbury agent 1	NA	cattle	NA	Pisoniviricetes	Picornavirales	Caliciviridae	Vertebrates
**NC_016994**	Night heron coronavirus HKU19	HKU19-6918	night-heron	NA	Pisoniviricetes	Nidovirales	Cornidovirineae	Vertebrates
**NC_038302**	Nilaparvata lugens honeydew virus 1	Izumo	Nilaparvata lugens	NA	Pisoniviricetes	Picornavirales	Iflaviridae	Invertebrates
**NC_021566**	Nilaparvata lugens honeydew virus-2	Izumo	Nilaparvata lugens	NA	Pisoniviricetes	Picornavirales	Iflaviridae	Invertebrates

**NC_021567**	Nilaparvata lugens honeydew virus-3	Kagoshima	Nilaparvata lugens	NA	Pisoniviricetes	Picornavirales	Iflaviridae	Invertebrates
**NC_032107**	NL63-related bat coronavirus	BtKYNL63-9a	Triaenops afer	NA	Pisoniviricetes	Nidovirales	Cornidovirineae	Vertebrates
**NC_048216**	NL63-related bat coronavirus	BtKYNL63-9b	Triaenops afer	NA	Pisoniviricetes	Nidovirales	Cornidovirineae	Vertebrates
**NC_055585**	Noni mosaic virus	NoMV-YJh	Morinda citrifolia	NA	Stelpaviricetes	Patatavirales	Potyviridae	Plants
**NC_044047**	Norovirus dog/GVI.1/HKU_Ca026F/2007/HKG	dog/GVI.1/HKU_Ca026F/2007/HKG	Canis lupus familiaris	NA	Pisoniviricetes	Picornavirales	Caliciviridae	Vertebrates
**NC_044856**	Norovirus GI	Hu/BD/2011/GI.7[PNA2]/Dhaka1882	Homo sapiens	NA	Pisoniviricetes	Picornavirales	Caliciviridae	Vertebrates
**NC_044853**	Norovirus GI	Hu/JP/1998/GI.6[PNA4]/No20-Saitama-98-17	Homo sapiens	NA	Pisoniviricetes	Picornavirales	Caliciviridae	Vertebrates
**NC_044854**	Norovirus GI	Hu/JP/2000/GI.6[PNA1]/WUG1	Homo sapiens	NA	Pisoniviricetes	Picornavirales	Caliciviridae	Vertebrates
**NC_001959**	Norovirus GI	NA	NA	NA	Pisoniviricetes	Picornavirales	Caliciviridae	Vertebrates
**NC_039897**	Norovirus GI/Hu/JP/2007/GI.P3_GI.3/Shimizu/KK2866	Shimizu/KK2866	Homo sapiens	NA	Pisoniviricetes	Picornavirales	Caliciviridae	Vertebrates
**NC_044932**	Norovirus GII	Hu/GII.PNA4-GII.NA4/PNV06929/2008/PER	Homo sapiens	NA	Pisoniviricetes	Picornavirales	Caliciviridae	Vertebrates
**NC_029646**	Norwalk-like virus	Hu/Norovirus/hiroshima/1999/JP(9912-02F)	NA	NA	Pisoniviricetes	Picornavirales	Caliciviridae	Vertebrates
**NC_039477**	Norovirus GII	NORO_226_06_01_2016	Homo sapiens	NA	Pisoniviricetes	Picornavirales	Caliciviridae	Vertebrates
**NC_039475**	Norovirus GII.17	Hu/GII.P17_GII.17/KR/2015/CAU-267	Homo sapiens	NA	Pisoniviricetes	Picornavirales	Caliciviridae	Vertebrates
**NC_039476**	Norovirus GII.2	BJSMQ	NA	NA	Pisoniviricetes	Picornavirales	Caliciviridae	Vertebrates
**NC_040876**	Norovirus GII	NORO_176-1_17_12_2015	Homo sapiens	NA	Pisoniviricetes	Picornavirales	Caliciviridae	Vertebrates
**NC_044045**	Norovirus GII/Hu/JP/2007/GII.P15_GII.15/Sapporo/HK299	Sapporo/HK299	Homo sapiens	NA	Pisoniviricetes	Picornavirales	Caliciviridae	Vertebrates
**NC_044046**	Norovirus GII/Hu/JP/2011/GII/Yuzawa/Gira2HS	Yuzawa/Gira2HS	Homo sapiens	NA	Pisoniviricetes	Picornavirales	Caliciviridae	Vertebrates
**NC_029645**	Norovirus GIII	Norovirus Bo/GIII.2/Adam/2006/No	bovine	NA	Pisoniviricetes	Picornavirales	Caliciviridae	Vertebrates
**NC_045762**	Norovirus GIV	CU081210E/USA/2010	Felis catus	NA	Pisoniviricetes	Picornavirales	Caliciviridae	Vertebrates
**NC_044855**	Norovirus GIV	Hu/US/2016/GIV.NA1[PNA1]/WI7002	Homo sapiens	NA	Pisoniviricetes	Picornavirales	Caliciviridae	Vertebrates
**NC_029647**	Norovirus GIV	Hu/GIV.1/LakeMacquarie/NSW268O/2010/AU	Homo sapiens	NA	Pisoniviricetes	Picornavirales	Caliciviridae	Vertebrates
**NC_008311**	Norovirus GV	Mu/NoV/GV/MNV1/2002/USA	NA	NA	Pisoniviricetes	Picornavirales	Caliciviridae	Vertebrates
**NC_025675**	hunnivirus A4	NrHuV/NYC-E21	Rattus norvegicus	NA	Pisoniviricetes	Picornavirales	Picornaviridae	Vertebrates
**NC_020901**	Alphamesonivirus 4	F24/CI/2004	Culex nebulosus	NA	Pisoniviricetes	Nidovirales	Mesnidovirineae	Invertebrates
**NC_030651**	Nylanderia fulva virus 1	Florida initial	Nylanderia fulva	NA	Pisoniviricetes	Picornavirales	Solinviviridae	Invertebrates
**NC_004016**	Oat mosaic virus	Cranbrook:laboratory isolate	oats	RNA 1	Stelpaviricetes	Patatavirales	Potyviridae	Plants
**NC_004017**	Oat mosaic virus	Cranbrook:laboratory isolate	oats	RNA 2	Stelpaviricetes	Patatavirales	Potyviridae	Plants
**NC_005136**	Oat necrotic mottle virus	Type-NE	NA	NA	Stelpaviricetes	Patatavirales	Potyviridae	Plants
**NC_043489**	Ofaie virus	BrMS-MQ10	Mansonia sp.	NA	Pisoniviricetes	Nidovirales	Mesnidovirineae	Invertebrates
**NC_038863**	Olive latent ringspot virus	Olive	Olive	NA	Pisoniviricetes	Picornavirales	Secoviridae	Plants
**NC_035127**	Olivier's shrew virus 1	Gkd-1	Crocidura olivieri guineensis	NA	Pisoniviricetes	Nidovirales	Arnidovirineae	Vertebrates
**NC_027917**	Opsiphanes invirae iflavirus 1	Brazilian/2012	Opsiphanes invirae (Lepidoptera: Nymphalidae)	NA	Pisoniviricetes	Picornavirales	Iflaviridae	Invertebrates
**NC_019409**	Ornithogalum mosaic virus	KP	NA	NA	Stelpaviricetes	Patatavirales	Potyviridae	Plants
**NC_002469**	Mamastrovirus 13		sheep	NA	Stelpaviricetes	Stellavirales	Astroviridae	Vertebrates
**NC_014252**	Panax virus Y	2	Panax notoginseng	NA	Stelpaviricetes	Patatavirales	Potyviridae	Plants
**NC_005028**	Papaya leaf distortion mosaic virus	NA	NA	NA	Stelpaviricetes	Patatavirales	Potyviridae	Plants
**NC_018449**	Papaya lethal yellowing virus	26	Carica papaya	NA	Pisoniviricetes	Sobelivirales	Solemoviridae	Plants
**NC_001785**	Papaya ringspot virus	NA	NA	NA	Stelpaviricetes	Patatavirales	Potyviridae	Plants
**NC_043532**	Paris mosaic necrosis virus	PMNV-cn	Daiswa polyphylla	NA	Stelpaviricetes	Patatavirales	Potyviridae	Plants
**NC_055600**	Paris virus 1	KM	Paris polyphylla var. yunnanensis	NA	Stelpaviricetes	Patatavirales	Potyviridae	Plants

**NC_003628**	Parsnip yellow fleck virus	P121	NA	NA	Pisoniviricetes	Picornavirales	Secoviridae	Plants
**NC_036588**	Passerivirus sp.	waxbill/DB01/HUN/2014	Uraeginthus granatina	NA	Pisoniviricetes	Picornavirales	Picornaviridae	Vertebrates
**NC_055497**	Passiflora edulis symptomless virus	PESV-Rehovot	Passiflora edulis f. edulis Sims cultivar Passion Dream	NA	Stelpaviricetes	Patatavirales	Potyviridae	Plants
**NC_014790**	Passion fruit woodiness virus	PWV-MU2	Passiflora caerulea	NA	Stelpaviricetes	Patatavirales	Potyviridae	Plants
**NC_003629**	Pea enation mosaic virus 1	WSG	NA	NA	Pisoniviricetes	Sobelivirales	Solemoviridae	Plants
**NC_001671**	Pea seed-borne mosaic virus	DPD1	NA	NA	Stelpaviricetes	Patatavirales	Potyviridae	Plants
**NC_034214**	Peach rosette mosaic virus	PRMV2	grape	RNA 1	Pisoniviricetes	Picornavirales	Secoviridae	Plants
**NC_034215**	Peach rosette mosaic virus	PRMV2	grape	RNA 2	Pisoniviricetes	Picornavirales	Secoviridae	Plants
**NC_002600**	Peanut mottle virus	NA	NA	NA	Stelpaviricetes	Patatavirales	Potyviridae	Plants
**NC_027124**	Pebjah virus	I621	rhesus macaque	NA	Pisoniviricetes	Nidovirales	Arnidovirineae	Vertebrates
**NC_030293**	Pecan mosaic-associated virus	LA	Carya illinoensis	NA	Stelpaviricetes	Patatavirales	Potyviridae	Plants
**NC_040673**	tremovirus B1	CNSR2011	Pelodiscus sinensis	NA	Pisoniviricetes	Picornavirales	Picornaviridae	Vertebrates
**NC_039004**	Penguin megrivirus	KGI-Bel-P5/2015	Pygoscelis adeliae	NA	Pisoniviricetes	Picornavirales	Picornaviridae	Vertebrates
**NC_028468**	Penicillium aurantiogriseum foetidus-like virus	NA	Penicillium aurantiogriseum isolate MUT4330-b	NA	Yadokarivirales	Yadokariviridae	Alphayadokarivirus	Fungi
**NC_007147**	Pennisetum mosaic virus	B	NA	NA	Stelpaviricetes	Patatavirales	Potyviridae	Plants
**NC_030225**	Pepo aphid-borne yellows virus	RSA BB Marrow	Cucurbita pepo L. (marrow)	NA	Pisoniviricetes	Sobelivirales	Solemoviridae	Plants
**NC_037052**	Pepper enamovirus	R1	Capsicum sp.	NA	Pisoniviricetes	Sobelivirales	Solemoviridae	Plants
**NC_001517**	Pepper mottle virus	NA	NA	NA	Stelpaviricetes	Patatavirales	Potyviridae	Plants
**NC_008393**	Pepper severe mosaic virus	NA	NA	NA	Stelpaviricetes	Patatavirales	Potyviridae	Plants
**NC_055129**	Pepper vein yellows virus 2	Is	Capsicum annuum	NA	Pisoniviricetes	Sobelivirales	Solemoviridae	Plants
**NC_036803**	Pepper vein yellows virus 5	Spain-Almeria 2-2013	Capsicum annuum cv. California Wonder	NA	Pisoniviricetes	Sobelivirales	Solemoviridae	Plants
**NC_015050**	Pepper vein yellows virus	NA	Capsicum annuum L. Var. grossum Sendt.	NA	Pisoniviricetes	Sobelivirales	Solemoviridae	Plants
**NC_011918**	Pepper veinal mottle virus	P	NA	NA	Stelpaviricetes	Patatavirales	Potyviridae	Plants
**NC_014327**	Pepper yellow mosaic virus	Pi-15	Capsicum annuum cv. Magali	NA	Stelpaviricetes	Patatavirales	Potyviridae	Plants
**NC_003113**	Perina nuda virus	NA	NA	NA	Pisoniviricetes	Picornavirales	Iflaviridae	Invertebrates
**NC_004573**	Peru tomato mosaic virus	PPK13	NA	NA	Stelpaviricetes	Patatavirales	Potyviridae	Plants
**NC_033492**	Petunia chlorotic mottle virus	Brats01	Petunia x hybrida	RNA 1	Pisoniviricetes	Picornavirales	Secoviridae	Plants
**NC_033493**	Petunia chlorotic mottle virus	Brats01	Petunia x hybrida	RNA 2	Pisoniviricetes	Picornavirales	Secoviridae	Plants
**NC_028793**	Phasey bean mild yellows virus	NSWCP15	chickpea	NA	Pisoniviricetes	Sobelivirales	Solemoviridae	Plants
**NC_027818**	Phopivirus	NewEngland_USA/2011	Phoca vitulina	NA	Pisoniviricetes	Picornavirales	Picornaviridae	Vertebrates
**NC_055577**	Physalis rugose mosaic virus	Piracicaba	Physalis peruviana	NA	Pisoniviricetes	Sobelivirales	Solemoviridae	Plants
**NC_034568**	Picorna-like virus AWando15	AWando15	Haliotis discus hannai (Pacific abalone)	NA	Pisoniviricetes	Picornavirales		Invertebrates
**NC_030697**	Picornavirales Bu-3	Bu-3	Sus scrofa domesticus	NA	Pisoniviricetes	Picornavirales		Vertebrates
**NC_030744**	Picornavirales Tottori-HG1	Tottori-HG1	Sus scrofa domesticus	NA	Pisoniviricetes	Picornavirales		Vertebrates
**NC_038989**	Picornaviridae sp. rodent/Ee/PicoV/NX2015	rodent/Ee/PicoV/NX2015	rodent	NA	Pisoniviricetes	Picornavirales	Picornaviridae	Vertebrates
**NC_038957**	pigeon mesivirus 1	HK21	pigeon	NA	Pisoniviricetes	Picornavirales	Picornaviridae	Vertebrates
**NC_015626**	Pigeon picornavirus B	03/641	Feral pigeon	NA	Pisoniviricetes	Picornavirales	Picornaviridae	Unknown
**NC_009020**	Pipistrellus bat coronavirus HKU5	HKU5-1 LMH03f	bat	NA	Pisoniviricetes	Nidovirales	Cornidovirineae	Vertebrates
**NC_034217**	Pityohyphantes rubrofasciatus iflavirus	UW1	Pityohyphantes rubrofasciatus	NA	Pisoniviricetes	Picornavirales	Iflaviridae	Invertebrates
**NC_055503**	Platycodon mild mottle virus	Okcheon	Platycodon grandiflorus	NA	Stelpaviricetes	Patatavirales	Potyviridae	Plants
**NC_003779**	Plautia stali intestine virus	NA	Plautia stali	NA	Pisoniviricetes	Picornavirales	Dicistroviridae	Invertebrates
**NC_040650**	Pleione flower breaking virus	CZ-Wharf1	Pleione humilis	NA	Stelpaviricetes	Patatavirales	Potyviridae	Plants
**NC_001445**	Plum pox virus	PPV-NAT	NA	NA	Stelpaviricetes	Patatavirales	Potyviridae	Plants

**NC_055108**	Poecivirus BCCH-449	BCCH-449	Black-capped chickadee	NA	Pisoniviricetes	Picornavirales	Picornaviridae	Vertebrates
**NC_011543**	Poinsettia latent virus	NA	Euphorbia pulcherrima cv. Angelika	NA	Pisoniviricetes	Sobelivirales	Solemoviridae	Plants
**NC_018872**	Pokeweed mosaic virus	PkMV-PA	Phytolacca americana L.	NA	Stelpaviricetes	Patatavirales	Potyviridae	Plants
**NC_002058**	Enterovirus C	Human poliovirus 1 Mahoney	NA	NA	Pisoniviricetes	Picornavirales	Picornaviridae	Vertebrates
**NC_038319**	Human parechovirus 1	NA	NA	NA	Pisoniviricetes	Picornavirales	Picornaviridae	Vertebrates
**NC_023674**	Porcine astrovirus 2	43/USA	Sus scrofa	NA	Stelpaviricetes	Stellavirales	Astroviridae	Vertebrates
**NC_019494**	Porcine astrovirus 3	US-MO123	Sus scrofa (swine)	NA	Stelpaviricetes	Stellavirales	Astroviridae	Vertebrates
**NC_023675**	Porcine astrovirus 4	35/USA	Sus scrofa	NA	Stelpaviricetes	Stellavirales	Astroviridae	Vertebrates
**NC_023636**	Porcine astrovirus 5	AstV5-US-IA122	Sus scrofa (swine)	NA	Stelpaviricetes	Stellavirales	Astroviridae	Vertebrates
**NC_039208**	Porcine coronavirus HKU15	HKU15-155	pig	NA	Pisoniviricetes	Nidovirales	Cornidovirineae	Vertebrates
**NC_004441**	Porcine enterovirus 9	UKG/410/73	NA	NA	Pisoniviricetes	Picornavirales	Picornaviridae	Vertebrates
**NC_003436**	Porcine epidemic diarrhea virus	CV777	NA	NA	Pisoniviricetes	Nidovirales	Cornidovirineae	Vertebrates
**NC_027054**	Porcine kobuvirus	JS-02a-CHN/2014/China	asymptomatic piglets	NA	Pisoniviricetes	Picornavirales	Picornaviridae	Vertebrates
**NC_016769**	Porcine kobuvirus SH-W-CHN/2010/China	SH-W-CHN/2010/China	pig	NA	Pisoniviricetes	Picornavirales	Picornaviridae	Vertebrates
**NC_011829**	Porcine kobuvirus swine/S-1-HUN/2007/Hungary	swine/S-1-HUN/2007/Hungary	Sus scrofa (domestic pig)	NA	Pisoniviricetes	Picornavirales	Picornaviridae	Vertebrates
**NC_038291**	Porcine reproductive and respiratory syndrome virus 2	ATCC VR-2332	Sus scrofa	NA	Pisoniviricetes	Nidovirales	Arnidovirineae	Vertebrates
**NC_001961**	Porcine reproductive and respiratory syndrome virus	NA	NA	NA	Pisoniviricetes	Nidovirales	Arnidovirineae	Vertebrates
**NC_003987**	Porcine sapelovirus 1	V13	NA	NA	Pisoniviricetes	Picornavirales	Picornaviridae	Vertebrates
**NC_003985**	Teschovirus A	F65	NA	NA	Pisoniviricetes	Picornavirales	Picornaviridae	Vertebrates
**NC_022787**	Porcine torovirus	SH1	porcine	NA	Pisoniviricetes	Nidovirales	Tornidovirineae	Vertebrates
**NC_023637**	Posavirus 1	NA	Sus scrofa	NA	Pisoniviricetes	Picornavirales	Posavirus	Unknown
**NC_023638**	Posavirus 2	NA	Sus scrofa	NA	Pisoniviricetes	Picornavirales	Posavirus	Unknown
**NC_028240**	Posavirus 3	958-4	swine	NA	Pisoniviricetes	Picornavirales	Posavirus	Unknown
**NC_008714**	enterovirus F4	W1	NA	NA	Pisoniviricetes	Picornavirales	Picornaviridae	Vertebrates
**NC_022798**	Potato black ringspot virus	PRI-Ec	Solanum tuberosum	RNA 1	Pisoniviricetes	Picornavirales	Secoviridae	Plants
**NC_001747**	Potato leafroll virus	NA	NA	NA	Pisoniviricetes	Sobelivirales	Solemoviridae	Plants
**NC_004039**	Potato virus A	infectious cDNA clone	potato (Solanum tuberosum)	NA	Stelpaviricetes	Patatavirales	Potyviridae	Plants
**NC_043447**	Potato virus B	Pasco-01	potato	RNA1	Pisoniviricetes	Picornavirales	Secoviridae	Plants
**NC_040417**	Potato virus U	UC	Solanum tuberosum ssp. andigena	RNA 1	Pisoniviricetes	Picornavirales	Secoviridae	Plants
**NC_040416**	Potato virus U	UC	Solanum tuberosum ssp. andigena	RNA 2	Pisoniviricetes	Picornavirales	Secoviridae	Plants
**NC_004010**	Potato virus V	DV 42	NA	NA	Stelpaviricetes	Patatavirales	Potyviridae	Plants
**NC_001616**	Potato virus Y	NA	NA	NA	Stelpaviricetes	Patatavirales	Potyviridae	Plants
**NC_031324**	Primate norovirus	SimianNoV-nj	NA	NA	Pisoniviricetes	Picornavirales	Caliciviridae	Vertebrates
**NC_039078**	Prunus virus F	8816-s1	sweet cherry	RNA2	Pisoniviricetes	Picornavirales	Secoviridae	Plants
**NC_039077**	Prunus virus F	8816-v1	sweet cherry	RNA1	Pisoniviricetes	Picornavirales	Secoviridae	Plants
**NC_040724**	Psammotettix alienus iflavirus 1	NA	Psammotettix alienus	NA	Pisoniviricetes	Picornavirales	Iflaviridae	Invertebrates
**NC_055513**	Pumpkin polerovirus	PuPV	Pumpkin sp.	NA	Pisoniviricetes	Sobelivirales	Solemoviridae	Plants
**NC_033792**	Qinghai Himalayan marmot astrovirus 1	HHMAstV1	Marmota himalayana	NA	Stelpaviricetes	Stellavirales	Astroviridae	Vertebrates
**NC_033821**	Qinghai Himalayan marmot astrovirus 2	HHMAstV2	Marmota himalayana	NA	Stelpaviricetes	Stellavirales	Astroviridae	Vertebrates
**NC_016403**	Quail picornavirus QPV1/HUN/2010	QPV1/HUN/2010	Coturnix coturnix (quail)	NA	Pisoniviricetes	Picornavirales	Picornaviridae	Vertebrates
**NC_025346**	Rabbit astrovirus TN/2208/2010	TN rabbit 10-2208	Oryctolagus cuniculus	NA	Stelpaviricetes	Stellavirales	Astroviridae	Vertebrates
**NC_011704**	Rabbit calicivirus Australia 1 MIC-07	MIC-07	rabbit	NA	Pisoniviricetes	Picornavirales	Caliciviridae	Vertebrates
**NC_017083**	Rabbit coronavirus HKU14	HKU14-1	Oryctolagus cuniculus	NA	Pisoniviricetes	Nidovirales	Cornidovirineae	Vertebrates
**NC_001543**	Rabbit hemorrhagic disease virus	FRG	NA	NA	Pisoniviricetes	Picornavirales	Caliciviridae	Vertebrates

**NC_026314**	rabbit kobuvirus	Berlin/Jan2011/0572	Rattus norvegicus	NA	Pisoniviricetes	Picornavirales	Picornaviridae	Vertebrates
**NC_039211**	Rabbit picornavirus	Ny4H/2010/HUN	Oryctolagus cuniculus var. domestica (domestic rabbit)	NA	Pisoniviricetes	Picornavirales	Picornaviridae	Vertebrates
**NC_008580**	Rabbit vesivirus	NA	Oryctolagus cuniculus	NA	Pisoniviricetes	Picornavirales	Caliciviridae	Vertebrates
**NC_055156**	Rabovirus B1	RtMp-PicoV/YN2014	rodent	NA	Pisoniviricetes	Picornavirales	Picornaviridae	Vertebrates
**NC_055160**	Rabovirus D1	MPV/NYC/2014/M005/0074	Mus musculus	NA	Pisoniviricetes	Picornavirales	Picornaviridae	Vertebrates
**NC_010709**	Radish mosaic virus	Japanese	NA	RNA 1	Pisoniviricetes	Picornavirales	Secoviridae	Plants
**NC_010710**	Radish mosaic virus	Japanese	NA	RNA 2	Pisoniviricetes	Picornavirales	Secoviridae	Plants
**NC_005267**	Raspberry ringspot virus	cherry	grapevine	RNA 2	Pisoniviricetes	Picornavirales	Secoviridae	Plants
**NC_005266**	Raspberry ringspot virus	cherry	grapevine	RNA 1	Pisoniviricetes	Picornavirales	Secoviridae	Plants
**NC_028963**	Rat arterivirus 1	Jilin2014	Myodes rufocanus	NA	Pisoniviricetes	Nidovirales	Arnidovirineae	Vertebrates
**NC_032987**	Rat arterivirus 1	Ningxia2015	Cricetulus longicaudatus	NA	Pisoniviricetes	Nidovirales	Arnidovirineae	Vertebrates
**NC_012936**	Rat coronavirus Parker	Parker	rat	NA	Pisoniviricetes	Nidovirales	Cornidovirineae	Vertebrates
**NC_003741**	Red clover mottle virus	S	NA	RNA 1	Pisoniviricetes	Picornavirales	Secoviridae	Plants
**NC_003738**	Red clover mottle virus	NA	NA	RNA 2	Pisoniviricetes	Picornavirales	Secoviridae	Plants
**NC_040399**	Red clover nepovirus A	B46	Trifolium pratense L.	RNA 1	Pisoniviricetes	Picornavirales	Secoviridae	Plants
**NC_040400**	Red clover nepovirus A	B46	Trifolium pratense L.	RNA 2	Pisoniviricetes	Picornavirales	Secoviridae	Plants
**NC_035461**	Reed chlorotic stripe virus	Tianshui	common reed	NA	Stelpaviricetes	Patatavirales	Potyviridae	Plants
**NC_040642**	Rhimavirus A	AU1	Rhinella marina	NA	Pisoniviricetes	Picornavirales	Picornaviridae	Vertebrates
**NC_009988**	Rhinolophus bat coronavirus HKU2	HKU2/GD/430/2006	NA	NA	Pisoniviricetes	Nidovirales	Cornidovirineae	Vertebrates
**NC_001490**	rhinovirus B14	NA	NA	NA	Pisoniviricetes	Picornavirales	Picornaviridae	Vertebrates
**NC_018613**	Rhizosolenia setigera RNA virus 01	RsRNAV06	NA	NA	Pisoniviricetes	Picornavirales	Marnaviridae	Chromista
**NC_001874**	Rhopalosiphum padi virus	NA	NA	NA	Pisoniviricetes	Picornavirales	Dicistroviridae	Invertebrates
**NC_028144**	Rice necrosis mosaic virus	NA	Oryza sativa	RNA 1	Stelpaviricetes	Patatavirales	Potyviridae	Plants
**NC_028145**	Rice necrosis mosaic virus	Ka-1	Oryza sativa	RNA 2	Stelpaviricetes	Patatavirales	Potyviridae	Plants
**NC_001632**	Rice tungro spherical virus	NA	NA	NA	Pisoniviricetes	Picornavirales	Secoviridae	Plants
**NC_001575**	Rice yellow mottle virus	CI4	NA	NA	Pisoniviricetes	Sobelivirales	Solemoviridae	Plants
**NC_031750**	Riptortus pedestris virus-1	1	Riptortus pedestris	NA	Pisoniviricetes	Picornavirales		Invertebrates
**NC_048210**	Rodent arterivirus	RtEi-Arterivirus/SX2014	Eothenomys inez	NA	Pisoniviricetes	Nidovirales	Arnidovirineae	Vertebrates
**NC_040535**	Rodent arterivirus	RtMc-Arterivirus/Tibet2014	Neodon clarkei	NA	Pisoniviricetes	Nidovirales	Arnidovirineae	Vertebrates
**NC_036583**	Rodent astrovirus	GX-006	Rattus norvegicus	NA	Stelpaviricetes	Stellavirales	Astroviridae	Vertebrates
**NC_046954**	Rodent coronavirus	RtMruf-CoV-2/JL2014	Myodes rufocanus	NA	Pisoniviricetes	Nidovirales	Cornidovirineae	Vertebrates
**NC_038314**	Rodent hepatovirus CIV459Lopsik2004	CIV459Lopsik2004	Lophuromys sikapusi	NA	Pisoniviricetes	Picornavirales	Picornaviridae	Vertebrates
**NC_038315**	Rodent hepatovirus KEF121Sigmas2012	KEF121Sigmas2012	Sigmodon mascotensis	NA	Pisoniviricetes	Picornavirales	Picornaviridae	Vertebrates
**NC_028363**	Rodent hepatovirus RMU101637Micarv2010	RMU101637Micarv2010	Microtus arvalis	NA	Pisoniviricetes	Picornavirales	Picornaviridae	Vertebrates
**NC_024070**	Rosavirus A2	GA7403	Homo sapiens	NA	Pisoniviricetes	Picornavirales	Picornaviridae	Vertebrates
**NC_031105**	Rosavirus B	RNCW0602091R	Rattus norvegicus	NA	Pisoniviricetes	Picornavirales	Picornaviridae	Vertebrates
**NC_031106**	Rosavirus C	RATLC11A	Rattus andamanensis	NA	Pisoniviricetes	Picornavirales	Picornaviridae	Vertebrates
**NC_038880**	Rosavirus M-7	Rosa.M-7	Peromyscus crinitus	NA	Pisoniviricetes	Picornavirales	Picornaviridae	Vertebrates
**NC_019031**	Rose yellow mosaic virus	Minnesota	Rosa hybrid cultivar cv. Ballerina	NA	Stelpaviricetes	Patatavirales	Potyviridae	Plants
**NC_027198**	Rottboellia yellow mottle virus	NA	NA	NA	Pisoniviricetes	Sobelivirales	Solemoviridae	Plants
**NC_018871**	Rousettus bat coronavirus HKU10	183A	bat	NA	Pisoniviricetes	Nidovirales	Cornidovirineae	Vertebrates
**NC_009021**	Rousettus bat coronavirus HKU9	HKU9-1 BF_005I	bat	NA	Pisoniviricetes	Nidovirales	Cornidovirineae	Vertebrates

**NC_030886**	Rousettus bat coronavirus	GCCDC1 356	Rousettus leschenaulti	NA	Pisoniviricetes	Nidovirales	Cornidovirineae	Vertebrates
**NC_011187**	Sowbane mosaic virus	NA	Rubus sp.	NA	Pisoniviricetes	Sobelivirales	Solemoviridae	Plants
**NC_001814**	Ryegrass mosaic virus	NA	NA	NA	Stelpaviricetes	Patatavirales	Potyviridae	Plants
**NC_003747**	Ryegrass mottle virus	MAFF. No. 307043	NA	NA	Pisoniviricetes	Sobelivirales	Solemoviridae	Plants
**NC_002066**	Sacbrood virus	Rothamstead	NA	NA	Pisoniviricetes	Picornavirales	Iflaviridae	Invertebrates
**NC_009448**	Saffold virus	NA	NA	NA	Pisoniviricetes	Picornavirales	Picornaviridae	Vertebrates
**NC_036802**	Saffron latent virus	Ir-Kh1	Crocus sativus L.	NA	Stelpaviricetes	Patatavirales	Potyviridae	Plants
**NC_012986**	Salivirus A	02394-01	Homo sapiens	NA	Pisoniviricetes	Picornavirales	Picornaviridae	Vertebrates
**NC_025114**	Salivirus FHB	SaliV-FHB	Homo sapiens; child	NA	Pisoniviricetes	Picornavirales	Picornaviridae	Vertebrates
**NC_012957**	Salivirus NG-J1	NG-J1	Homo sapiens	NA	Pisoniviricetes	Picornavirales	Picornaviridae	Vertebrates
**NC_025676**	San Miguel sea lion virus 8	NA	NA	NA	Pisoniviricetes	Picornavirales	Caliciviridae	Vertebrates
**NC_006554**	Sapovirus C12	C12	NA	NA	Pisoniviricetes	Picornavirales	Caliciviridae	Vertebrates
**NC_006269**	Sapovirus Hu/Dresden/pJG-Sap01/DE	Hu/Dresden/pJG-Sap01/DE	NA	NA	Pisoniviricetes	Picornavirales	Caliciviridae	Vertebrates
**NC_027026**	Sapovirus Hu/Nagoya/NGY-1/2012/JPN	Hu/SaV/Nagoya/NGY-1/2012/JPN	NA	NA	Pisoniviricetes	Picornavirales	Caliciviridae	Vertebrates
**NC_010624**	Sapovirus Mc10	Mc10	NA	NA	Pisoniviricetes	Picornavirales	Caliciviridae	Vertebrates
**NC_004718**	SARS coronavirus Tor2	Tor2	Homo sapiens	NA	Pisoniviricetes	Nidovirales	Cornidovirineae	Vertebrates
**NC_003785**	Satsuma dwarf virus	S-58	NA	RNA 1	Pisoniviricetes	Picornavirales	Secoviridae	Plants
**NC_003786**	Satsuma dwarf virus	S-58	NA	RNA 2	Pisoniviricetes	Picornavirales	Secoviridae	Plants
**NC_025837**	Sauropus yellowing virus	THSP1-B	Sauropus androgynus	NA	Pisoniviricetes	Sobelivirales	Solemoviridae	Plants
**NC_003399**	Scallion mosaic virus	NA	Allium chinense	NA	Stelpaviricetes	Patatavirales	Potyviridae	Plants
**NC_007522**	Aurantiochytrium single-stranded RNA virus 01	NA	NA	NA	Pisoniviricetes	Picornavirales	Marnaviridae	Chromista
**NC_009657**	Scotophilus bat coronavirus 512	BtCoV/512/2005	NA	NA	Pisoniviricetes	Nidovirales	Cornidovirineae	Vertebrates
**NC_009891**	Seal picornavirus type 1	HO.02.21	Phoca hispida	NA	Pisoniviricetes	Picornavirales	Picornaviridae	Vertebrates
**NC_021482**	Sebokele virus 1	1	Hylomyscus	NA	Pisoniviricetes	Picornavirales	Picornaviridae	Vertebrates
**NC_011349**	Senecavirus A	SVV-001	NA	NA	Pisoniviricetes	Picornavirales	Picornaviridae	Vertebrates
**NC_002568**	Sesbania mosaic virus	NA	NA	NA	Pisoniviricetes	Sobelivirales	Solemoviridae	Plants
**NC_045512**	Severe acute respiratory syndrome coronavirus 2	Wuhan-Hu-1	Homo sapiens	NA	Pisoniviricetes	Nidovirales	Cornidovirineae	Vertebrates
**NC_007433**	Shallot yellow stripe virus	ZQ2	Allium fistulosum	NA	Stelpaviricetes	Patatavirales	Potyviridae	Plants
**NC_038961**	Shanbavirus A	BtMf-PicoV-1/SAX2011	Miniopterus fuliginosus	NA	Pisoniviricetes	Picornavirales	Picornaviridae	Vertebrates
**NC_046955**	Shrew coronavirus	Shrew-CoV/Tibet2014	Sorex araneus	NA	Pisoniviricetes	Nidovirales	Cornidovirineae	Vertebrates
**NC_028364**	Shrew hepatovirus KS121232Sorara2012	KS121232Sorara2012	Sorex araneus	NA	Pisoniviricetes	Picornavirales	Picornaviridae	Vertebrates
**NC_032978**	Shuangao insect virus 8	insectZJ96360	insects	NA	Pisoniviricetes	Picornavirales	Polycipiviridae	Invertebrates
**NC_037655**	Sichuan takin astrovirus	LLT03	Budorcas taxicolor tibetana	NA	Stelpaviricetes	Stellavirales	Astroviridae	Vertebrates
**NC_037654**	Sichuan takin enterovirus	LLT03	Budorcas taxicolor tibetana	NA	Pisoniviricetes	Picornavirales	Picornaviridae	Vertebrates
**NC_023861**	Sicinivirus A	UCC001	chicken	NA	Pisoniviricetes	Picornavirales	Picornaviridae	Vertebrates
**NC_028380**	sicinivirus A1	JSY	chicken	NA	Pisoniviricetes	Picornavirales	Picornaviridae	Vertebrates
**NC_003988**	Enterovirus H	NA	NA	NA	Pisoniviricetes	Picornavirales	Picornaviridae	Vertebrates
**NC_038309**	Simian enterovirus SV4	1715 UWB	NA	NA	Pisoniviricetes	Picornavirales	Picornaviridae	Vertebrates
**NC_038293**	Simian hemorrhagic encephalitis virus	Sukhumi	Macaca mulatta	NA	Pisoniviricetes	Nidovirales	Arnidovirineae	Vertebrates
**NC_003092**	Simian hemorrhagic fever virus	LVR 42-0/M6941	NA	NA	Pisoniviricetes	Nidovirales	Arnidovirineae	Vertebrates
**NC_004451**	Simian sapelovirus 1	2383	NA	NA	Pisoniviricetes	Picornavirales	Picornaviridae	Vertebrates
**NC_014137**	Slow bee paralysis virus	Rothamsted	NA	NA	Pisoniviricetes	Picornavirales	Iflaviridae	Invertebrates
**NC_033706**	Solanum nodiflorum mottle virus	NA	Solanum nodiflorum	NA	Pisoniviricetes	Sobelivirales	Solemoviridae	Plants
**NC_006559**	Solenopsis invicta virus 1	NA	Solenopsis invicta	NA	Pisoniviricetes	Picornavirales	Dicistroviridae	Invertebrates
**NC_039236**	Solenopsis invicta virus 2	Florida-Sin	Solenopsis invicta (ant)	NA	Pisoniviricetes	Picornavirales	Polycipiviridae	Invertebrates
**NC_012531**	Solenopsis invicta virus 3	DM	Solenopsis invicta (red imported fire ant)	NA	Pisoniviricetes	Picornavirales	Solinviviridae	Invertebrates
**NC_035455**	Solenopsis invicta virus 4	Gainesville-Sin	Solenopsis invicta (ant)	NA	Pisoniviricetes	Picornavirales	Polycipiviridae	Invertebrates

**NC_004035**	Sorghum mosaic virus	Xiaoshan	sugarcane	NA	Stelpaviricetes	Patatavirales	Potyviridae	Plants
**NC_004060**	Southern bean mosaic virus	Sao Paulo	NA	NA	Pisoniviricetes	Sobelivirales	Solemoviridae	Plants
**NC_025113**	Southwest baboon virus 1	SWBV_16986_11/4/2013	Papio anubis	NA	Pisoniviricetes	Nidovirales	Arnidovirineae	Vertebrates
**NC_032270**	Soybean latent spherical virus	ND1	soybean	RA11	Pisoniviricetes	Picornavirales	Secoviridae	Plants
**NC_032271**	Soybean latent spherical virus	ND1	soybean	RA22	Pisoniviricetes	Picornavirales	Secoviridae	Plants
**NC_002634**	Soybean mosaic virus	N	NA	NA	Stelpaviricetes	Patatavirales	Potyviridae	Plants
**NC_016033**	Soybean yellow common mosaic virus	NA	Glycine max	NA	Pisoniviricetes	Sobelivirales	Solemoviridae	Plants
**NC_016992**	Sparrow coronavirus HKU17	HKU17-6124	sparrow	NA	Pisoniviricetes	Nidovirales	Cornidovirineae	Vertebrates
**NC_016405**	Spodoptera exigua iflavirus 1	NA	Spodoptera exigua	NA	Pisoniviricetes	Picornavirales	Iflaviridae	Invertebrates
**NC_023676**	Spodoptera exigua iflavirus 2	Korean	Spodoptera exigua	NA	Pisoniviricetes	Picornavirales	Iflaviridae	Invertebrates
**NC_035221**	Squash chlorotic leaf spot virus	Su12-10	Cucurbita moschata (squash)	RNA 1	Pisoniviricetes	Picornavirales	Secoviridae	Plants
**NC_035215**	Squash chlorotic leaf spot virus	Su12-10	Cucurbita moschata (squash)	RNA 2	Pisoniviricetes	Picornavirales	Secoviridae	Plants
**NC_003799**	Squash mosaic virus	Y-SqMV	NA	RNA 1	Pisoniviricetes	Picornavirales	Secoviridae	Plants
**NC_003800**	Squash mosaic virus	Y-SqMV	NA	RNA 2	Pisoniviricetes	Picornavirales	Secoviridae	Plants
**NC_010521**	Squash vein yellowing virus	Florida	watermelon	NA	Stelpaviricetes	Patatavirales	Potyviridae	Plants
**NC_011050**	Steller sea lion vesivirus	SSL2004-250F	NA	NA	Pisoniviricetes	Picornavirales	Caliciviridae	Vertebrates
**NC_006964**	Strawberry latent ringspot virus	NCGR MEN 454.001	NA	RNA 1	Pisoniviricetes	Picornavirales	Secoviridae	Plants
**NC_003445**	Strawberry mottle virus	NA	NA	NA	Pisoniviricetes	Picornavirales	Secoviridae	Plants
**NC_018571**	Suakwa aphid-borne yellows virus	SABYV-TW19	Luffa cylindrica (Suakwa vegetable sponge)	NA	Pisoniviricetes	Sobelivirales	Solemoviridae	Plants
**NC_004346**	Subterranean clover mottle virus	p23	Trifolium subterraneum	NA	Pisoniviricetes	Sobelivirales	Solemoviridae	Plants
**NC_035459**	Sudan watermelon mosaic virus	Su94-54	cucumis melo var. flexuosus	NA	Stelpaviricetes	Patatavirales	Potyviridae	Plants
**NC_003398**	Sugarcane mosaic virus	NA	maize	NA	Stelpaviricetes	Patatavirales	Potyviridae	Plants
**NC_014037**	Sugarcane streak mosaic virus	PAK	Saccharum hybrid	NA	Stelpaviricetes	Patatavirales	Potyviridae	Plants
**NC_000874**	Sugarcane yellow leaf virus	A	Saccharum sp. cultivar CP65-357	NA	Pisoniviricetes	Sobelivirales	Solemoviridae	Plants
**NC_014038**	Sunflower chlorotic mottle virus	Common (C)	Helianthus annuus	NA	Stelpaviricetes	Patatavirales	Potyviridae	Plants
**NC_021065**	Sunflower mild mosaic virus	Entre Rios	Helianthus annuus (sunflower)	NA	Stelpaviricetes	Patatavirales	Potyviridae	Plants
**NC_034208**	Sunflower ring blotch virus	Chaco	Helianthus annuus	NA	Stelpaviricetes	Patatavirales	Potyviridae	Plants
**NC_001841**	Sweet potato feathery mottle virus	S	NA	NA	Stelpaviricetes	Patatavirales	Potyviridae	Plants
**NC_020896**	Sweet potato latent virus	NA	Ipomoea batatas	NA	Stelpaviricetes	Patatavirales	Potyviridae	Plants
**NC_038510**	Sweet potato leaf speckling virus	Peruvian	NA	NA	Pisoniviricetes	Sobelivirales	Solemoviridae	Plants
**NC_003797**	Sweet potato mild mottle virus	NA	NA	NA	Stelpaviricetes	Patatavirales	Potyviridae	Plants
**NC_038563**	Sweet potato mild speckling virus	NA	NA	NA	Stelpaviricetes	Patatavirales	Potyviridae	Plants
**NC_017970**	Sweet potato virus 2	GWB-2	Ipomoea batatas cv. Beauregard	NA	Stelpaviricetes	Patatavirales	Potyviridae	Plants
**NC_014742**	Sweet potato virus C	C1	NA	NA	Stelpaviricetes	Patatavirales	Potyviridae	Plants
**NC_018093**	Sweet potato virus G	Jesus Maria	Ipomoea batatas cv. Arapey	NA	Stelpaviricetes	Patatavirales	Potyviridae	Plants
**NC_028806**	Swine enteric coronavirus	Italy/213306/2009	pig	NA	Pisoniviricetes	Nidovirales	Cornidovirineae	Vertebrates
**NC_018226**	Pasivirus A1	NA	Sus scrofa	NA	Pisoniviricetes	Picornavirales	Picornaviridae	Vertebrates
**NC_055159**	tottorivirus A1	Tottori-WOL	Sus scrofa domesticus	NA	Pisoniviricetes	Picornavirales	Picornaviridae	Vertebrates
**NC_022745**	Tall oatgrass mosaic virus	Benesov	Arrhenatherum elatius	NA	Stelpaviricetes	Patatavirales	Potyviridae	Plants
**NC_026615**	Tamarillo leaf malformation virus	A	Solanum betaceum	NA	Stelpaviricetes	Patatavirales	Potyviridae	Plants
**NC_003005**	Taura syndrome virus	NA	Penaeus vannamei	NA	Pisoniviricetes	Picornavirales	Dicistroviridae	Invertebrates
**NC_009742**	Telosma mosaic virus	Hanoi	Telosma cordata (telosma)	NA	Stelpaviricetes	Patatavirales	Potyviridae	Plants
**NC_026250**	Thaumetopoea pityocampa iflavirus 1	NA	Thaumetopoea pityocampa	NA	Pisoniviricetes	Picornavirales	Iflaviridae	Invertebrates

**NC_001366**	Theilovirus	GDVII	NA	NA	Pisoniviricetes	Picornavirales	Picornaviridae	Vertebrates
**NC_011549**	Thrush coronavirus HKU12-600	NA	grey-backed thrush	NA	Pisoniviricetes	Nidovirales	Cornidovirineae	Vertebrates
**NC_007180**	Thunberg fritillary mosaic virus	Ningbo	Fritillaria thunbergii	NA	Stelpaviricetes	Patatavirales	Potyviridae	Plants
**NC_001555**	Tobacco etch virus	NA	NA	NA	Stelpaviricetes	Patatavirales	Potyviridae	Plants
**NC_030118**	Tobacco mosqueado virus	RS-01	Nicotiana tabacum (tobacco)	NA	Stelpaviricetes	Patatavirales	Potyviridae	Plants
**NC_005097**	Tobacco ringspot virus	Bud Blight	NA	RNA 1	Pisoniviricetes	Picornavirales	Secoviridae	Plants
**NC_005096**	Tobacco ringspot virus	NA	NA	RNA 2	Pisoniviricetes	Picornavirales	Secoviridae	Plants
**NC_009994**	Tobacco vein banding mosaic virus	YND	tobacco	NA	Stelpaviricetes	Patatavirales	Potyviridae	Plants
**NC_010732**	Tobacco vein distorting virus	Longlin	tobacco	NA	Pisoniviricetes	Sobelivirales	Solemoviridae	Plants
**NC_001768**	Tobacco vein mottling virus	NA	NA	NA	Stelpaviricetes	Patatavirales	Potyviridae	Plants
**NC_034265**	Tobacco virus 2	TV2	Nicotiana tabacum	NA	Pisoniviricetes	Sobelivirales	Solemoviridae	Plants
**NC_004439**	Tomato black ring virus	MJ	NA	RNA 1	Pisoniviricetes	Picornavirales	Secoviridae	Plants
**NC_004440**	Tomato black ring virus	MJ	NA	RNA 2	Pisoniviricetes	Picornavirales	Secoviridae	Plants
**NC_013075**	Tomato chocolate spot virus	NA	tomato	RNA 1	Pisoniviricetes	Picornavirales	Secoviridae	Plants
**NC_013076**	Tomato chocolate spot virus	NA	tomato	RNA2	Pisoniviricetes	Picornavirales	Secoviridae	Plants
**NC_010987**	Tomato marchitez virus	PRI-TMarV0601	NA	RNA 1	Pisoniviricetes	Picornavirales	Secoviridae	Plants
**NC_010988**	Tomato marchitez virus	PRI-TMarV0601	NA	RNA 2	Pisoniviricetes	Picornavirales	Secoviridae	Plants
**NC_038920**	Tomato mild mottle virus	NA	NA	NA	Stelpaviricetes	Patatavirales	Potyviridae	Plants
**NC_027926**	Tomato necrotic dwarf virus	R	Solanum lycopersicum (tomato)	RNA1	Pisoniviricetes	Picornavirales	Secoviridae	Plants
**NC_027927**	Tomato necrotic dwarf virus	R	Solanum lycopersicum (tomato)	RNA2	Pisoniviricetes	Picornavirales	Secoviridae	Plants
**NC_017824**	Tomato necrotic stunt virus	MX9354	Solanum lycopersicum (tomato)	NA	Stelpaviricetes	Patatavirales	Potyviridae	Plants
**NC_003840**	Tomato ringspot virus	raspberry	NA	RNA 1	Pisoniviricetes	Picornavirales	Secoviridae	Plants
**NC_003839**	Tomato ringspot virus	NA	NA	RNA 2	Pisoniviricetes	Picornavirales	Secoviridae	Plants
**NC_009032**	Tomato torrado virus	PRI-ToTV0301	NA	RNA 2	Pisoniviricetes	Picornavirales	Secoviridae	Plants
**NC_025890**	torchivirus A1	44665	Testudo hermanni	NA	Pisoniviricetes	Picornavirales	Picornaviridae	Vertebrates
**NC_023988**	Tortoise rafivirus A	UF4	Indotestudo forsteni	NA	Pisoniviricetes	Picornavirales	Picornaviridae	Vertebrates
**NC_038861**	Transmissible gastroenteritis virus	PUR46-MAD	pig	NA	Pisoniviricetes	Nidovirales	Cornidovirineae	Vertebrates
**NC_003783**	Triatoma virus	NA	Triatoma infestans	NA	Pisoniviricetes	Picornavirales	Dicistroviridae	Invertebrates
**NC_012799**	Triticum mosaic virus	U06-123	Triticum aestivum cultivar RonL	NA	Stelpaviricetes	Patatavirales	Potyviridae	Plants
**NC_043424**	Tuberose mild mottle virus	Hangzhou	NA	NA	Stelpaviricetes	Patatavirales	Potyviridae	Plants
**NC_043512**	Tulane virus	NA	NA	NA	Pisoniviricetes	Picornavirales	Caliciviridae	Vertebrates
**NC_028981**	Tupaia hepatovirus A	TN1	Tupaia belangeri chinensis	NA	Pisoniviricetes	Picornavirales	Picornaviridae	Vertebrates
**NC_014411**	Passerivirus A1	356	Pale Thrush	NA	Pisoniviricetes	Picornavirales	Picornaviridae	Vertebrates
**NC_014412**	Oscivirus A1	10717	Oriental Magpie Robin	NA	Pisoniviricetes	Picornavirales	Picornaviridae	Vertebrates
**NC_014413**	Oscivirus A2	10878	Grey-backed Thrush	NA	Pisoniviricetes	Picornavirales	Picornaviridae	Vertebrates
**NC_005790**	Turkey astrovirus 2	NA	NA	NA	Stelpaviricetes	Stellavirales	Astroviridae	Vertebrates
**NC_002470**	Turkey astrovirus		turkey	NA	Stelpaviricetes	Stellavirales	Astroviridae	Vertebrates
**NC_038304**	Turkey avisivirus	USA-IN1	Meleagris gallopavo	NA	Pisoniviricetes	Picornavirales	Picornaviridae	Vertebrates
**NC_043516**	Turkey calicivirus	L11043	turkey	NA	Pisoniviricetes	Picornavirales	Caliciviridae	Vertebrates
**NC_010800**	Turkey coronavirus	MG10	turkey	NA	Pisoniviricetes	Nidovirales	Cornidovirineae	Vertebrates
**NC_021201**	Turkey hepatitis virus 2993D	124	turkey poult	NA	Pisoniviricetes	Picornavirales	Picornaviridae	Vertebrates
**NC_002509**	Turnip mosaic virus	NA	NA	NA	Stelpaviricetes	Patatavirales	Potyviridae	Plants
**NC_013218**	Turnip ringspot virus	Toledo	NA	RNA 1	Pisoniviricetes	Picornavirales	Secoviridae	Plants
**NC_013219**	Turnip ringspot virus	Toledo	NA	RNA 2	Pisoniviricetes	Picornavirales	Secoviridae	Plants
**NC_003743**	Turnip yellows virus	FL1	NA	NA	Pisoniviricetes	Sobelivirales	Solemoviridae	Plants
**NC_009019**	Tylonycteris bat coronavirus HKU4	HKU4-1 B04f	bat	NA	Pisoniviricetes	Nidovirales	Cornidovirineae	Vertebrates

**NC_014791**	Ugandan cassava brown streak virus	UG	Manihot esculenta	NA	Stelpaviricetes	Patatavirales	Potyviridae	Plants
**NC_029992**	Free State vervet virus	VSAI1003	Chlorocebus sp.	NA	Pisoniviricetes	Nidovirales	Arnidovirineae	Vertebrates
**NC_017977**	Cyrtanthus elatus virus A	Marijiniup 7	Cyrtanthus elatus	NA	Stelpaviricetes	Patatavirales	Potyviridae	Plants
**NC_025250**	Vanilla distortion mosaic virus	VDMV-Cor	Coriandrum sativum	NA	Stelpaviricetes	Patatavirales	Potyviridae	Plants
**NC_040601**	Varroa destructor virus 2	VDV-2	Varroa destructor (mite)	NA	Pisoniviricetes	Picornavirales	Iflaviridae	Invertebrates
**NC_006494**	Varroa destructor virus 1	NA	Varroa destructor mites	NA	Pisoniviricetes	Picornavirales	Iflaviridae	Invertebrates
**NC_014509**	Velvet tobacco mottle virus	K1	Nicotiana velutina	NA	Pisoniviricetes	Sobelivirales	Solemoviridae	Plants
**NC_010735**	Verbena virus Y	Michigan	Verbena sp. cv. 'Taylor Town'	NA	Stelpaviricetes	Patatavirales	Potyviridae	Plants
**NC_002551**	Vesicular exanthema of swine virus	NA	NA	NA	Pisoniviricetes	Picornavirales	Caliciviridae	Vertebrates
**NC_027122**	Vesivirus ferret badger/JX12/China/2012	FBCV-JX12	ferret badger	NA	Pisoniviricetes	Picornavirales	Caliciviridae	Vertebrates
**NC_004541**	Walrus calicivirus	NA	walrus	NA	Pisoniviricetes	Picornavirales	Caliciviridae	Vertebrates
**NC_030843**	Washington bat picornavirus	UW1	NA	NA	Pisoniviricetes	Picornavirales	Picornaviridae	Invertebrates
**NC_006262**	Watermelon mosaic virus	WMV-Fr	NA	NA	Stelpaviricetes	Patatavirales	Potyviridae	Plants
**NC_035191**	Wencheng Sm shrew coronavirus	Xingguo-101	Suncus murinus	NA	Pisoniviricetes	Nidovirales	Cornidovirineae	Vertebrates
**NC_009805**	Wheat eqlid mosaic virus	NA	NA	NA	Stelpaviricetes	Patatavirales	Potyviridae	Plants
**NC_035451**	Wheat leaf yellowing-associated virus	JN-U3	Triticum aestivum (wheat)	NA	Pisoniviricetes	Sobelivirales	Solemoviridae	Plants
**NC_040508**	Wheat spindle streak mosaic virus	WSSMV_South	NA	RNA 1	Stelpaviricetes	Patatavirales	Potyviridae	Plants
**NC_040507**	Wheat spindle streak mosaic virus	WSSMV_South	NA	RNA 2	Stelpaviricetes	Patatavirales	Potyviridae	Plants
**NC_001886**	Wheat streak mosaic virus	NA	NA	NA	Stelpaviricetes	Patatavirales	Potyviridae	Plants
**NC_012931**	Wheat yellow dwarf virus-GPV	NA	Triticum aestivum	NA	Pisoniviricetes	Sobelivirales	Solemoviridae	Plants
**NC_002350**	Wheat yellow mosaic virus	NA	NA	RNA 1	Stelpaviricetes	Patatavirales	Potyviridae	Plants
**NC_002349**	Wheat yellow mosaic virus	NA	NA	RNA 2	Stelpaviricetes	Patatavirales	Potyviridae	Plants
**NC_008516**	White bream virus	DF24/00	Blicca bjoerkna L.	NA	Pisoniviricetes	Nidovirales	Tornidovirineae	Vertebrates
**NC_031747**	White clover mottle virus	CD	Trifolium repens	NA	Pisoniviricetes	Sobelivirales	Solemoviridae	Plants
**NC_016991**	White-eye coronavirus HKU16	HKU16-6847	white-eye	NA	Pisoniviricetes	Nidovirales	Cornidovirineae	Vertebrates
**NC_016995**	Wigeon coronavirus HKU20	HKU20-9243	wigeon	NA	Pisoniviricetes	Nidovirales	Cornidovirineae	Vertebrates
**NC_035458**	Wild melon vein banding virus	Su03-07	Cucumis melo var. agrestis	NA	Stelpaviricetes	Patatavirales	Potyviridae	Plants
**NC_030391**	Wild onion symptomless virus	TUR256-1	Wild onion	NA	Stelpaviricetes	Patatavirales	Potyviridae	Plants
**NC_004426**	Wild potato mosaic virus	Type Isolate	NA	NA	Stelpaviricetes	Patatavirales	Potyviridae	Plants
**NC_009744**	Wild tomato mosaic virus	Laichau	Solanum torvum (wild tomato)	NA	Stelpaviricetes	Patatavirales	Potyviridae	Plants
**NC_007216**	Wisteria vein mosaic virus	Beijing	Wisteria	NA	Stelpaviricetes	Patatavirales	Potyviridae	Plants
**NC_026811**	Wobbly possum disease virus	WPDV	Trichosurus vulpecula (Australian brushtail possum)	NA	Pisoniviricetes	Nidovirales	Arnidovirineae	Vertebrates
**NC_046957**	Wuhan japanese halfbeak arterivirus	DSYS15584	Hyporhamphus sajori	NA	Pisoniviricetes	Nidovirales	Nanidovirineae	Vertebrates
**NC_043490**	Xinzhou nematode virus 6	XZSJSC61041	snake-associated nematodes	NA	Pisoniviricetes	Nidovirales	Tornidovirineae	Vertebrates
**NC_040360**	Yado-kari virus 1	2-W1032/S6	NA	NA	Yadokarivirales	Yadokariviridae	Alphayadokarivirus	Fungi
**NC_029854**	Yak enterovirus	SWUN-AB001	yaks	NA	Pisoniviricetes	Picornavirales	Picornaviridae	Vertebrates
**NC_016441**	Yambean mosaic virus	SR	yam bean	NA	Stelpaviricetes	Patatavirales	Potyviridae	Plants
**NC_055491**	Yam chlorotic necrosis virus	YCNV-YJish	Dioscorea sp.	NA	Stelpaviricetes	Patatavirales	Potyviridae	Plants
**NC_038561**	Yam chlorotic necrotic mosaic virus	YS	Dioscorea parviflora	NA	Stelpaviricetes	Patatavirales	Potyviridae	Plants
**NC_019412**	Yam mild mosaic virus	NA	Dioscorea trifida	NA	Stelpaviricetes	Patatavirales	Potyviridae	Plants
**NC_004752**	Yam mosaic virus	Ivory Coast	NA	NA	Stelpaviricetes	Patatavirales	Potyviridae	Plants
**NC_048215**	Yellow head virus	20120706	Fenneropenaeus chinensis	NA	Pisoniviricetes	Nidovirales	Ronidovirineae	Invertebrates

**NC_043505**	Yellow head virus	Chachoengsao 1998	Penaeus monodon	NA	Pisoniviricetes	Nidovirales	Ronidovirineae	Invertebrates
**NC_024471**	Yellow oat grass mosaic virus	YOgMV-Sb	Trisetum flavescens L. (Yellow oatgrass)	NA	Stelpaviricetes	Patatavirales	Potyviridae	Plants
**NC_040534**	Yichang virus	HB-MLV	Culex sp. (mosquito)	NA	Pisoniviricetes	Nidovirales	Mesnidovirineae	Invertebrates
**NC_040587**	Yongsan iflavirus 1	A16.2047/ROK/2016	Culex pipiens	NA	Pisoniviricetes	Picornavirales	Iflaviridae	Invertebrates
**NC_040584**	Yongsan picorna-like virus 3	YPLV3/16-0052/ROK/2016	Aedes vexans nipponii	NA	Pisoniviricetes	Picornavirales		Invertebrates
**NC_040654**	Yongsan picorna-like virus 4	16-0128/ROK/2016	Aedes vexans nipponii	NA	Pisoniviricetes	Picornavirales		Invertebrates
**NC_048209**	Zambian malbrouck virus 1	SHFVagmMal_seqID_01	Chlorocebus sp.	NA	Pisoniviricetes	Nidovirales	Arnidovirineae	Vertebrates
**NC_011560**	Zantedeschia mild mosaic virus	TW	Zantedeschia spp. (calla lily)	NA	Stelpaviricetes	Patatavirales	Potyviridae	Plants
**NC_043172**	Zucchini shoestring virus	RSA Patty pan	Cucurbita pepo	NA	Stelpaviricetes	Patatavirales	Potyviridae	Plants
**NC_023175**	Zucchini tigre mosaic virus	Re01-25	Momordica charantia	NA	Stelpaviricetes	Patatavirales	Potyviridae	Plants
**NC_003224**	Zucchini yellow mosaic virus	TW-TN3	Luffa cylindrica Roem.	NA	Stelpaviricetes	Patatavirales	Potyviridae	Plants
**AB006531**	Plautia stali intestine virus	NA	Plautia stali	NA	Pisoniviricetes	Picornavirales	Dicistroviridae	Invertebrates
**AF014388**	Drosophila C virus	EB	NA	NA	Pisoniviricetes	Picornavirales	Dicistroviridae	Invertebrates
**AF022937**	Rhopalosiphum padi virus	NA	NA	NA	Pisoniviricetes	Picornavirales	Dicistroviridae	Invertebrates
**AB000906**	Infectious flacherie virus	NA	NA	NA	Pisoniviricetes	Picornavirales	Iflaviridae	Invertebrates
**AB017037**	Himetobi P virus	NA	Laodelphax striatellus	NA	Pisoniviricetes	Picornavirales	Dicistroviridae	Invertebrates
**AF092924**	Sacbrood virus	Rothamstead	NA	NA	Pisoniviricetes	Picornavirales	Iflaviridae	Invertebrates
**AF178440**	Triatoma virus	NA	Triatoma infestans	NA	Pisoniviricetes	Picornavirales	Dicistroviridae	Invertebrates
**AF183905**	Black queen cell virus	South African	Apis mellifera	NA	Pisoniviricetes	Picornavirales	Dicistroviridae	Invertebrates
**AF218039**	Cricket paralysis virus	NA	NA	NA	Pisoniviricetes	Picornavirales	Dicistroviridae	Invertebrates
**AF150629**	Acute bee paralysis virus	NA	Apis mellifera	NA	Pisoniviricetes	Picornavirales	Dicistroviridae	Invertebrates
**AF277675**	Taura syndrome virus	NA	Penaeus vannamei	NA	Pisoniviricetes	Picornavirales	Dicistroviridae	Invertebrates
**AF323747**	Perina nuda virus	NA	NA	NA	Pisoniviricetes	Picornavirales	Iflaviridae	Invertebrates
**AF469603**	Sacbrood virus	NA	NA	NA	Pisoniviricetes	Picornavirales	Iflaviridae	Invertebrates
**AF536531**	Aphid lethal paralysis virus	NA	NA	NA	Pisoniviricetes	Picornavirales	Dicistroviridae	Invertebrates
**AY275710**	Kashmir bee virus	NA	NA	NA	Pisoniviricetes	Picornavirales	Dicistroviridae	Invertebrates
**AY292384**	Deformed wing virus	PA	NA	NA	Pisoniviricetes	Picornavirales	Iflaviridae	Invertebrates
**AY341824**	Ectropis obliqua picorna-like virus	NA	NA	NA	Pisoniviricetes	Picornavirales	Iflaviridae	Invertebrates
**AY152711S1**	Sacbrood virus	Riez2002	NA	NA	Pisoniviricetes	Picornavirales	Iflaviridae	Invertebrates
**AF486072**	Acute bee paralysis virus	Hungary 1	Apis mellifera	NA	Pisoniviricetes	Picornavirales	Dicistroviridae	Invertebrates
**AY365064**	Ectropis obliqua picorna-like virus	NA	Ectropis obliqua	NA	Pisoniviricetes	Picornavirales	Iflaviridae	Invertebrates
**AY224602**	Deformed wing virus	NA	NA	NA	Pisoniviricetes	Picornavirales	Iflaviridae	Invertebrates
**AB070959**	Kakugo virus	NA	Apis mellifera	NA	Pisoniviricetes	Picornavirales	Iflaviridae	Invertebrates
**AB183472**	Himetobi P virus	Izumo	NA	NA	Pisoniviricetes	Picornavirales	Dicistroviridae	Invertebrates
**AY590471**	Taura syndrome virus	Belize	NA	NA	Pisoniviricetes	Picornavirales	Dicistroviridae	Invertebrates
**AY634314**	Solenopsis invicta virus 1	NA	Solenopsis invicta	NA	Pisoniviricetes	Picornavirales	Dicistroviridae	Invertebrates
**AY251269**	Varroa destructor virus 1	NA	Varroa destructor mites	NA	Pisoniviricetes	Picornavirales	Iflaviridae	Invertebrates
**AY997025**	Taura syndrome virus	Th04Lv	Litopenaeus vannamei	NA	Pisoniviricetes	Picornavirales	Dicistroviridae	Invertebrates
**DQ104696**	Taura syndrome virus	ZHZC3TSV	Penaeus vannamei	NA	Pisoniviricetes	Picornavirales	Dicistroviridae	Invertebrates
**AJ489744**	Deformed wing virus	NA	Apis mellifera	NA	Pisoniviricetes	Picornavirales	Iflaviridae	Invertebrates
**DQ112227**	Kelp fly virus	NA	Chaetocoelopa sydneyensis	NA	Pisoniviricetes	Picornavirales		Invertebrates
**DQ212790**	Taura syndrome virus	NA	NA	NA	Pisoniviricetes	Picornavirales	Dicistroviridae	Invertebrates
**DQ288865**	Homalodisca coagulata virus 1	NA	NA	NA	Pisoniviricetes	Picornavirales	Dicistroviridae	Invertebrates
**DQ224278**	Deformed wing virus	AUSTRIA-1	NA	NA	Pisoniviricetes	Picornavirales	Iflaviridae	Invertebrates
**EF219380**	Israeli acute paralysis virus	NA	NA	NA	Pisoniviricetes	Picornavirales	Dicistroviridae	Invertebrates
**EF517277**	Brevicoryne brassicae virus - UK	NA	Brevicoryne brassicae	NA	Pisoniviricetes	Picornavirales	Iflaviridae	Invertebrates
**EF428566**	Solenopsis invicta virus 2	NA	Solenopsis invicta (fire ant)	NA	Pisoniviricetes	Picornavirales	Polycipiviridae	Invertebrates
**EF517505**	Black queen cell virus	AUSTRIA-5	NA	NA	Pisoniviricetes	Picornavirales	Dicistroviridae	Invertebrates
**EF517515**	Black queen cell virus	HUNGARY-10	NA	NA	Pisoniviricetes	Picornavirales	Dicistroviridae	Invertebrates
**EF517519**	Black queen cell virus	POLAND-4	NA	NA	Pisoniviricetes	Picornavirales	Dicistroviridae	Invertebrates

**EU035616**	Slow bee paralysis virus	Rothamsted	NA	NA	Pisoniviricetes	Picornavirales	Iflaviridae	Invertebrates
**EU436423**	Israeli acute paralysis virus	DVE31-OP3-PA-USA-2007	NA	NA	Pisoniviricetes	Picornavirales	Dicistroviridae	Invertebrates
**EU436455**	Israeli acute paralysis virus	IS1-ISRAEL-2007	NA	NA	Pisoniviricetes	Picornavirales	Dicistroviridae	Invertebrates
**EU436456**	Israeli acute paralysis virus	OZ6-AUSTRALIA-2007	NA	NA	Pisoniviricetes	Picornavirales	Dicistroviridae	Invertebrates
**EU868609**	Infectious flacherie virus	ZheJiang01/CHN	Bombyx mori	NA	Pisoniviricetes	Picornavirales	Iflaviridae	Invertebrates
**FJ790488**	Nasonia vitripennis virus	3	Nasonia vitripennis	NA	Pisoniviricetes	Picornavirales	Iflaviridae	Invertebrates
